# Correcting the Mean-Variance Dependency for Differential Variability Testing Using Single-Cell RNA Sequencing Data

**DOI:** 10.1016/j.cels.2018.06.011

**Published:** 2018-09-26

**Authors:** Nils Eling, Arianne C. Richard, Sylvia Richardson, John C. Marioni, Catalina A. Vallejos

**Affiliations:** 1 European Molecular Biology Laboratory, European Bioinformatics Institute (EMBL-EBI), Wellcome Genome Campus, Hinxton, Cambridge CB10 1SD, UK; 2 Cancer Research UK Cambridge Institute, University of Cambridge, Li Ka Shing Centre, Cambridge CB2 0RE, UK; 3 Cambridge Institute for Medical Research, University of Cambridge, Cambridge Biomedical Campus, Hills Road, Cambridge CB2 0XY, UK; 4 MRC Biostatistics Unit, University of Cambridge, Cambridge Institute of Public Health, Forvie Site, Robinson Way, Cambridge Biomedical Campus, Cambridge CB2 0SR, UK; 5 The Alan Turing Institute, British Library, 96 Euston Road, London NW1 2DB, UK; 6 Department of Statistical Science, University College London, 1-19 Torrington Place, London WC1E 7HB, UK; 7 MRC Human Genetics Unit, MRC Institute of Genetics and Molecular Medicine, University of Edinburgh, Western General Hospital, Crewe Road, Edinburgh EH4 2XY, UK

**Keywords:** single-cell RNA sequencing, transcriptional noise, variability, immune activation, statistics, Bayesian

## Abstract

Cell-to-cell transcriptional variability in otherwise homogeneous cell populations plays an important role in tissue function and development. Single-cell RNA sequencing can characterize this variability in a transcriptome-wide manner. However, technical variation and the confounding between variability and mean expression estimates hinder meaningful comparison of expression variability between cell populations. To address this problem, we introduce an analysis approach that extends the BASiCS statistical framework to derive a residual measure of variability that is not confounded by mean expression. This includes a robust procedure for quantifying technical noise in experiments where technical spike-in molecules are not available. We illustrate how our method provides biological insight into the dynamics of cell-to-cell expression variability, highlighting a synchronization of biosynthetic machinery components in immune cells upon activation. In contrast to the uniform up-regulation of the biosynthetic machinery, CD4^+^ T cells show heterogeneous up-regulation of immune-related and lineage-defining genes during activation and differentiation.

## Highlights

• Correction of the mean-variability dependency in scRNA-seq data using BASiCS
• Quantification of technical noise when spike-in RNA is missing
• Joint differential mean expression and differential variability testing
• Detection of heterogeneously regulated genes during immune responses

## Graphical Abstract

**Figure:**
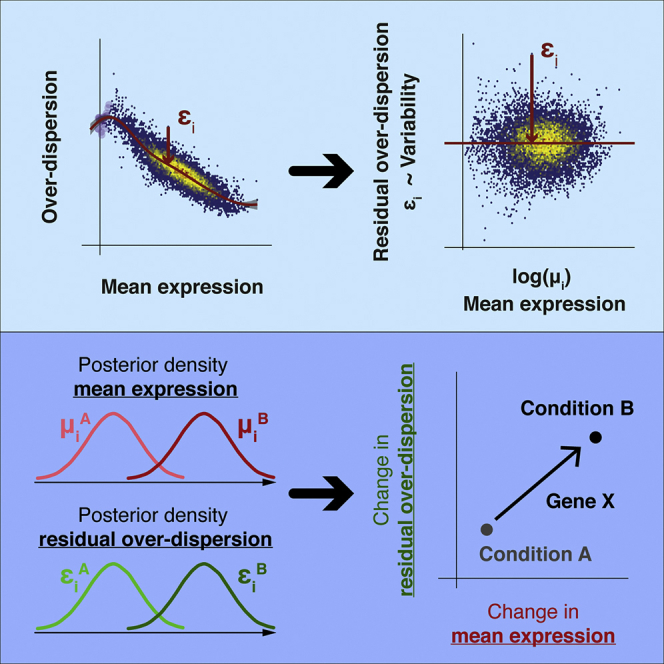

## Teaser

When quantifying expression variability from single-cell RNA sequencing data, lowly expressed genes tend to be more variable compared to highly expressed genes. This confounding effect hinders robust expression variability testing between two cell populations when mean expression changes. Eling et al. introduce a Bayesian approach that corrects for this confounding effect and allows joint differential mean and differential variability testing. This led to the discovery of homogeneously and heterogeneously regulated genes during immune activation and differentiation.

## Introduction

Heterogeneity in gene expression within a population of single cells can arise from a variety of factors. Structural differences in gene expression within a cell population can reflect the presence of sub-populations of functionally different cell types (Zeisel et al., 2015, Paul et al., 2015). Alternatively, in a seemingly homogeneous population of cells, the so-called unstructured expression heterogeneity can be linked to intrinsic or extrinsic noise (Elowitz et al., 2002). Changes in physiological cell states (such as cell cycle, metabolism, abundance of transcriptional and translational machinery, and growth rate) represent extrinsic noise, which has been found to influence expression variability within cell populations (Miller, 1981, Keren et al., 2015, Buettner et al., 2015, Zeng et al., 2017). Intrinsic noise can be linked to epigenetic diversity (Smallwood et al., 2014), chromatin rearrangements (Buenrostro et al., 2015), as well as the genomic context of single genes, such as the presence of TATA-box motifs and the abundance of nucleosomes around the transcriptional start site (Hornung et al., 2012).

Single-cell RNA sequencing (scRNA-seq) generates transcriptional profiles of single cells, allowing the study of cell-to-cell heterogeneity on a transcriptome-wide (Grün et al., 2014) and single gene level (Goolam et al., 2016). Consequently, this technique can be used to study unstructured cell-to-cell variation in gene expression within and between homogeneous cell populations (i.e., where no distinct cell sub-types are present). Increasing evidence suggests that this heterogeneity plays an important role in normal development (Chang et al., 2008) and that control of expression noise is important for tissue function (Bahar Halpern et al., 2015). For instance, molecular noise was shown to increase before cells commit to lineages during differentiation (Mojtahedi et al., 2016), while the opposite is observed once an irreversible cell state is reached (Richard et al., 2016). A similar pattern occurs during gastrulation, where expression noise is high in the uncommitted inner cell mass compared to the committed epiblast and where an increase in heterogeneity is observed when cells exit the pluripotent state and form the uncommitted epiblast (Mohammed et al., 2017).

Motivated by scRNA-seq, recent studies have extended traditional differential expression analyses to explore more general patterns that characterize differences between cell populations or experimental conditions (e.g., Korthauer et al. [2016]). In particular, the Bayesian analysis of single-cell sequencing data (BASiCS) framework (Vallejos et al., 2015, 2016) introduced a probabilistic tool to assess differences in cell-to-cell heterogeneity between two or more cell populations. This feature has led to, for example, insights into the context of immune activation and aging (Martinez-Jimenez et al., 2017, Miller, 1981). To meaningfully assess changes in biological variability across the entire transcriptome, two main confounding effects must be taken into account: differences due to artefactual technical noise and differential variability between populations that is driven by changes in mean expression. The latter arises because biological noise is negatively correlated with protein abundance (Bar-Even et al., 2006, Newman et al., 2006, Taniguchi et al., 2010) or mean RNA expression (Brennecke et al., 2013, Antolović et al., 2017). To address these two confounding effects, BASiCS separates biological noise from technical variability by borrowing information from synthetic RNA spike-in molecules. Additionally, to acknowledge the variance-mean relationship, it restricts differential variability testing to those genes with equal mean expression across populations.

This article extends the statistical model implemented in BASiCS by introducing a more general approach to account for the aforementioned confounding effects. First, we derive a residual measure of cell-to-cell transcriptional variability that is not confounded by mean expression. This is used to define a probabilistic rule to robustly highlight changes in variability, even for differentially expressed genes. Unlike previous related methods (e.g., Kolodziejczyk et al. [2015]), our approach directly performs gene-specific statistical testing between two conditions using a readily available measure of uncertainty. Second, by exploiting concepts from measurement error models, our method is extended to address experimental designs where spike-in sequences are not available. This is particularly critical due to the increasing popularity of droplet-based technologies.

Using our approach, we identify a synchronization of biosynthetic machinery components in CD4^+^ T cells upon early immune activation as well as an increased variability in the expression of genes related to CD4^+^ T cell immunological function. Furthermore, we detect evidence of early cell fate commitment of CD4^+^ T cells during malaria infection characterized by a decrease in *Tbx21* expression heterogeneity and a rapid collapse of global transcriptional variability after infection. These results highlight biological insights into T cell activation and differentiation that are only revealed by jointly studying changes in mean expression and variability.

## Results

### Addressing the Mean Confounding Effect for Differential Variability Testing

Unlike bulk RNA-seq, scRNA-seq provides information about cell-to-cell expression heterogeneity within a population of cells. Previous studies have used a variety of measures to quantify this heterogeneity. Among others, this includes the coefficient of variation (CV) (Brennecke et al., 2013) and entropy measures (Richard et al., 2016). As in Vallejos et al., 2015, Vallejos et al., 2016, we focus on biological *over-dispersion* as a proxy for transcriptional heterogeneity. This is defined by the excess of variability that is observed with respect to what would be predicted by Poisson sampling noise after accounting for technical variation.

The aforementioned measures of variability can be used to identify genes whose transcriptional heterogeneity differs between groups of cells (defined by experimental conditions or cell types). However, the strong relationship that is typically observed between variability and mean estimates (e.g., Brennecke et al. [2013]) can hinder the interpretation of these results.

**Figure 1:**
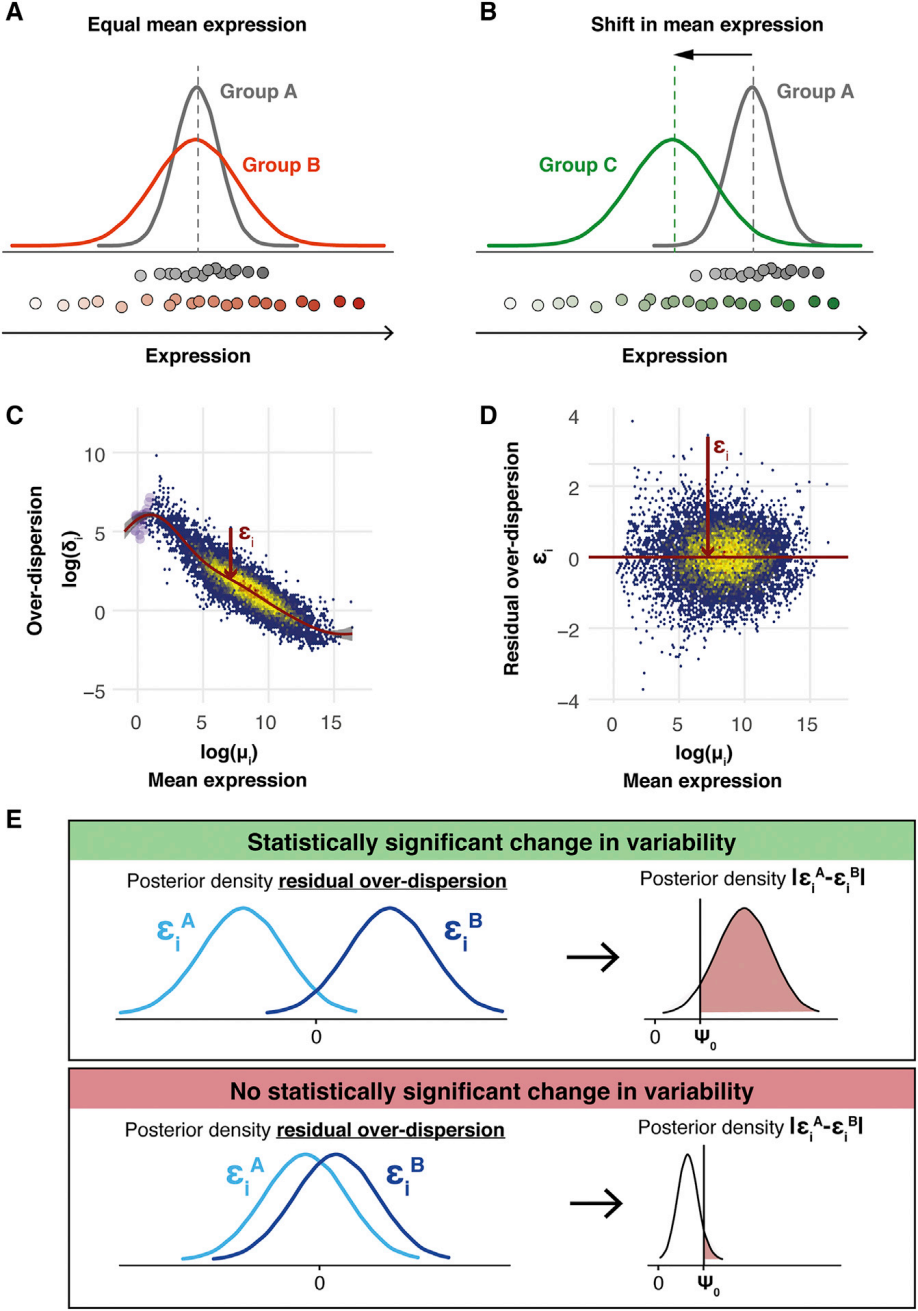
Avoiding the Mean Confounding Effect When Quantifying Expression Variability in scRNA-Seq Data (A and B) Illustration of changes in expression variability for a single gene between two cell populations without (A) and with (B) changes in mean expression. (C and D) Our extended BASiCS model infers a regression trend between gene-specific estimates of over-dispersion parameters *δ*_*i*_ and mean expression *μ*_*i*_. Residual over-dispersion parameters $\epsilon_{i}$ are defined by departures from the regression trend. For a single gene, this is illustrated using a red arrow. The color code within the scatterplots is used to represent areas with high (yellow and red) and low (blue) concentration of genes. For illustration purposes, the data introduced by Antolović et al. (2017) have been used (see STAR Methods). (C) Gene-specific estimates of over-dispersion parameters *δ*_*i*_ were plotted against mean expression parameters *μ*_*i*_. The red line shows the regression trend. This illustrates the typical confounding effect that is observed between variability and mean expression measures. Genes that are not detected in at least 2 cells are indicated by purple points. (D) Gene-specific estimates of residual over-dispersion parameters $\epsilon_{i}$ were plotted against mean expression parameters *μ*_*i*_. This illustrates the lack of correlation between these parameters. (E) Illustration of how posterior uncertainty is used to highlight changes in residual over-dispersion. Two example genes with (upper) and without (lower) differential residual over-dispersion are shown. Left inset illustrates the posterior density associated with residual over-dispersion parameters $\epsilon_{i}$ for a gene in two groups of cells (group A, light blue; group B, dark blue). The colored area in the right inset represents the posterior probability of observing an absolute difference $\left|\epsilon_{i}^{A}-\epsilon_{i}^{B}\right|$ that is larger than the minimum tolerance threshold *ψ*_0_ (see STAR Methods).

A simple solution to avoid this confounding is to restrict the assessment of differential variability to those genes with equal mean expression across populations (see Figure 1A). However, this is sub-optimal, particularly when a large number of genes are differentially expressed between the populations. For example, reactive genes that change in mean expression upon changing conditions (e.g., transcription factors) are excluded from differential variability testing. An alternative approach is to directly adjust variability measures to remove this confounding. For example, Kolodziejczyk et al. (2015) computed the empirical distance between the squared CV to a rolling median along expression levels—referred to as the DM method.

In line with this idea, our method extends the statistical model implemented in BASiCS (Vallejos et al., 2015, Vallejos et al., 2016). We define a measure of “residual over-dispersion”—which is not correlated with mean expression—to meaningfully assess changes in transcriptional heterogeneity when genes exhibit shifts in mean expression (see Figure 1B). More concretely, we infer a regression trend between over-dispersion (*δ*_*i*_) and gene-specific mean parameters (*μ*_*i*_), by introducing a joint informative prior to capture the dependence between these parameters (see STAR Methods). A latent gene-specific residual over-dispersion parameter $\epsilon_{i}$ describes departures from this trend (see Figure 1C). Positive values of $\epsilon_{i}$ indicate that a gene exhibits more variation than expected relative to genes with similar expression levels. Similarly, negative values of $\epsilon_{i}$ suggest less variation than expected, and, as shown in Figure 1D, these residual over-dispersion parameters are not confounded by mean expression.

Our hierarchical Bayes approach infers full posterior distributions for the gene-specific latent residual over-dispersion parameters $\epsilon_{i}$. As a result, we can directly use a probabilistic approach to identify genes with large absolute differences in residual over-dispersion between two groups of cells (see Figure 1E and STAR Methods). The performance of this differential variability test was validated using simulated data (see Figure S1 and STAR Methods). In contrast, mean-corrected point estimates for residual noise parameters (such as those obtained by the DM method) cannot be directly used to perform gene-specific statistical testing between two conditions, as no measure of the uncertainty in the estimate is readily available.

### The Informative Prior Stabilizes Parameter Estimation

Our joint prior formulation has introduced a non-linear regression to capture the overall trend between gene-specific over-dispersion parameters *δ*_*i*_ and mean expression parameters *μ*_*i*_ (see STAR Methods). Thus, we also refer to the extended model induced by this prior as the “regression” BASiCS model. Accordingly, the model induced by the original independent prior specification (Vallejos et al., 2016) is referred to as the “non-regression” BASiCS model.

**Figure 2:**
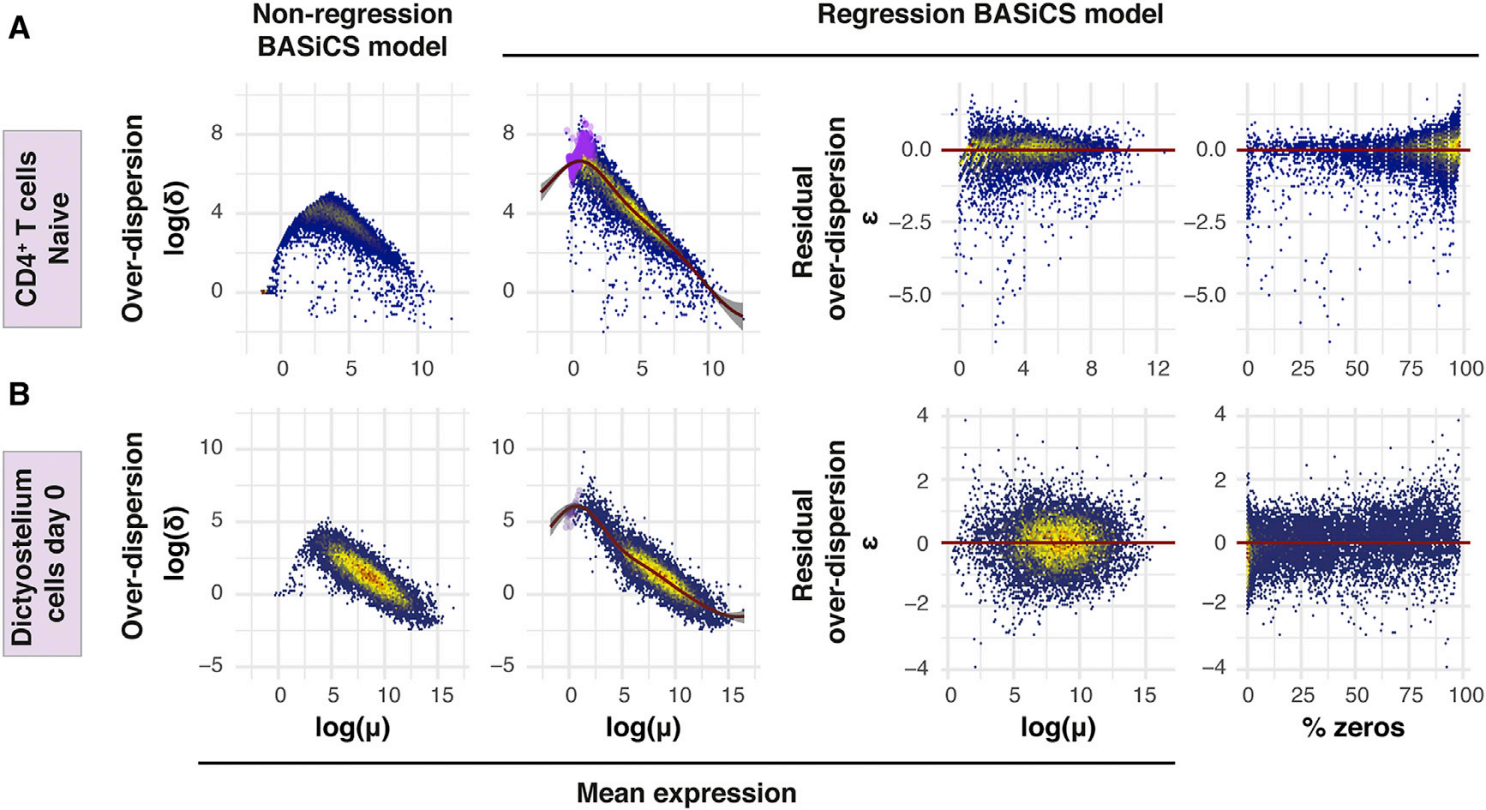
Parameter Estimation Using a Variety of scRNA-Seq Datasets Model parameters were estimated using the regression and non-regression BASiCS models on (A) naive CD4^+^ T cells (Martinez-Jimenez et al., 2017) and (B) *Dictyostelium* cells prior to differentiation (day 0) (Antolović et al., 2017). These datasets were selected to highlight two situations with different levels of sparsity (i.e., the proportion of zero counts; see fourth column). More details about these datasets are provided in STAR Methods. The color code within the scatterplots is used to represent areas with high (yellow and red) and low (blue) concentration of genes. First column: gene-specific over-dispersion *δ*_*i*_ versus mean expression *μ*_*i*_ as estimated by the non-regression BASiCS model. Second column: gene-specific over-dispersion *δ*_*i*_ versus mean expression *μ*_*i*_ as estimated by the regression BASiCS model. The red line indicates the estimated regression trend. Purple dots indicate genes detected (i.e., with at least one count) in fewer than 2 cells. Third column: gene-specific residual over-dispersion $\epsilon_{i}$ versus mean expression *μ*_*i*_ as estimated by the regression BASiCS model. Fourth column: gene-specific posterior estimates for residual over-dispersion $\epsilon_{i}$ parameters versus percentage of zero counts for each gene. See also Figure S2.

To study the performance of the regression BASiCS model, we applied it to a variety of scRNA-seq datasets. Each dataset is unique in its composition, covering a range of different cell types and experimental protocols (see STAR Methods and Table S1). Qualitatively, we observe that the inferred regression trend varies substantially across different datasets (Figures 2 and S2), justifying the choice of a flexible semi-parametric approach (see STAR Methods). Moreover, as expected, we observe that residual over-dispersion parameters $\epsilon_{i}$ are not confounded by mean expression nor by the percentage of zero counts per gene.

The regression BASiCS model introduces a joint prior specification for (*μ*_*i*_, *δ*_*i*_)′, shrinking the posterior estimates for *μ*_*i*_ and *δ*_*i*_ toward the regression trend (this is in line with the shrinkage observed in Love et al. [2014]). The strength of this shrinkage is dataset specific, being more prominent in sparser datasets with a higher frequency of zero counts (see Figure 2A) and for lowly expressed genes where measurement error is greatest.

**Figure 3:**
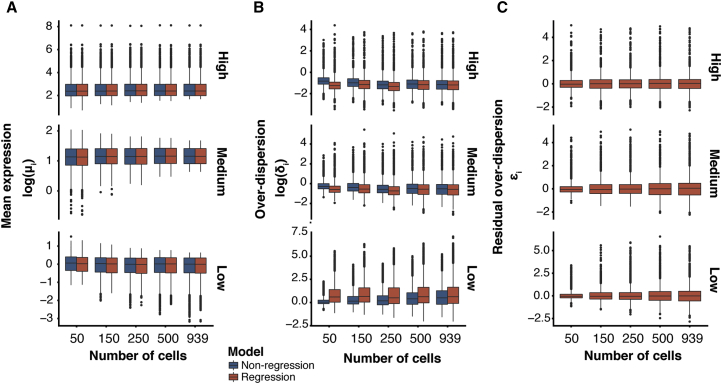
Estimation of Gene-Specific Model Parameters for Varying Sample Sizes The regression (orange) and non-regression (blue) BASiCS models were used to estimate gene-specific model parameters for lowly (lower insets), medium (mid insets), and highly (upper insets) expressed genes across populations with varying numbers of cells. These were generated by randomly sub-sampling cells from a population of 939 CA1 pyramidal neurons (Zeisel et al., 2015). For more details, see STAR Methods. Extended results based on multiple downsampling experiments are displayed in Figures S3D–S3F. (A–C) For a single sub-sampling experiment, boxplots summarize the distribution of gene-specific estimates for (A) mean expression parameters *μ*_*i*_ (log-scale), (B) over-dispersion parameters *δ*_*i*_ (log-scale), and (C) residual over-dispersion parameters $\epsilon_{i}$. See also Figure S3.

Subsequently, we asked whether or not the shrinkage introduced by the regression BASiCS model improves posterior inference. To assess this, we compared estimates for gene-specific parameters across (1) different sample sizes and (2) different gene expression levels. More concretely, we used a large dataset containing 939 CA1 pyramidal neurons (Zeisel et al., 2015) to artificially generate smaller datasets by randomly sub-sampling 50–500 cells. For each sample size, parameter estimates were then obtained using both the regression and non-regression BASiCS models. The distribution of these estimates is summarized in Figure 3.

First, we observe that both the regression and non-regression BASiCS models led to comparable and largely stable mean expression estimates across different sample sizes and expression levels (see Figure 3A). Second, in line with the results in Figure 2, the main differences between the methods arise when estimating the over-dispersion parameters δ_*i*_ (see Figures 3B and S3A–S3C). In particular, we observe that the non-regression BASiCS model appears to underestimate δ_*i*_ for lowly expressed genes when the sample size is small (with respect to the parameter estimates obtained based on the full dataset of 939 cells). In contrast, the shrinkage introduced by our regression BASiCS model aids parameter estimation, leading to robust estimates even for the smallest sample size. This is particularly important for rare cell populations where large sample sizes are difficult to obtain. A similar effect is observed for genes with medium and high expression levels, where the non-regression BASiCS model appears to overestimate δ_*i*_. We also observe that estimates of residual over-dispersion parameters $\epsilon_{i}$ are stable across sample sizes and expression levels. These findings are replicated across multiple sub-sampling experiments (see Figures S3D–S3F).

As an external validation, we compared our posterior estimates of gene-specific model parameters obtained from scRNA-seq data to empirical estimates from matched single molecule fluorescence *in situ* hybridization (smFISH) data of mouse embryonic stem cells grown in 2i and serum media (see STAR Methods and Grün et al. [2014]). First, posterior estimates of mean-expression parameters *μ*_*i*_ exhibit high correlation to smFISH mean transcript counts (see Figure S3G). Second, we also observe a strong correlation between posterior estimates for over-dispersion parameters *δ*_*i*_ and the empirical CV^2^ values obtained from smFISH data (see Figure S3H). Finally, a similar behavior is observed when comparing posterior estimates of residual over-dispersion parameters $\epsilon_{i}$ to a residual CV^2^ (see Figure S3I and STAR Methods).

### Inferring Technical Variability without Spike-In Genes

**Figure 4:**
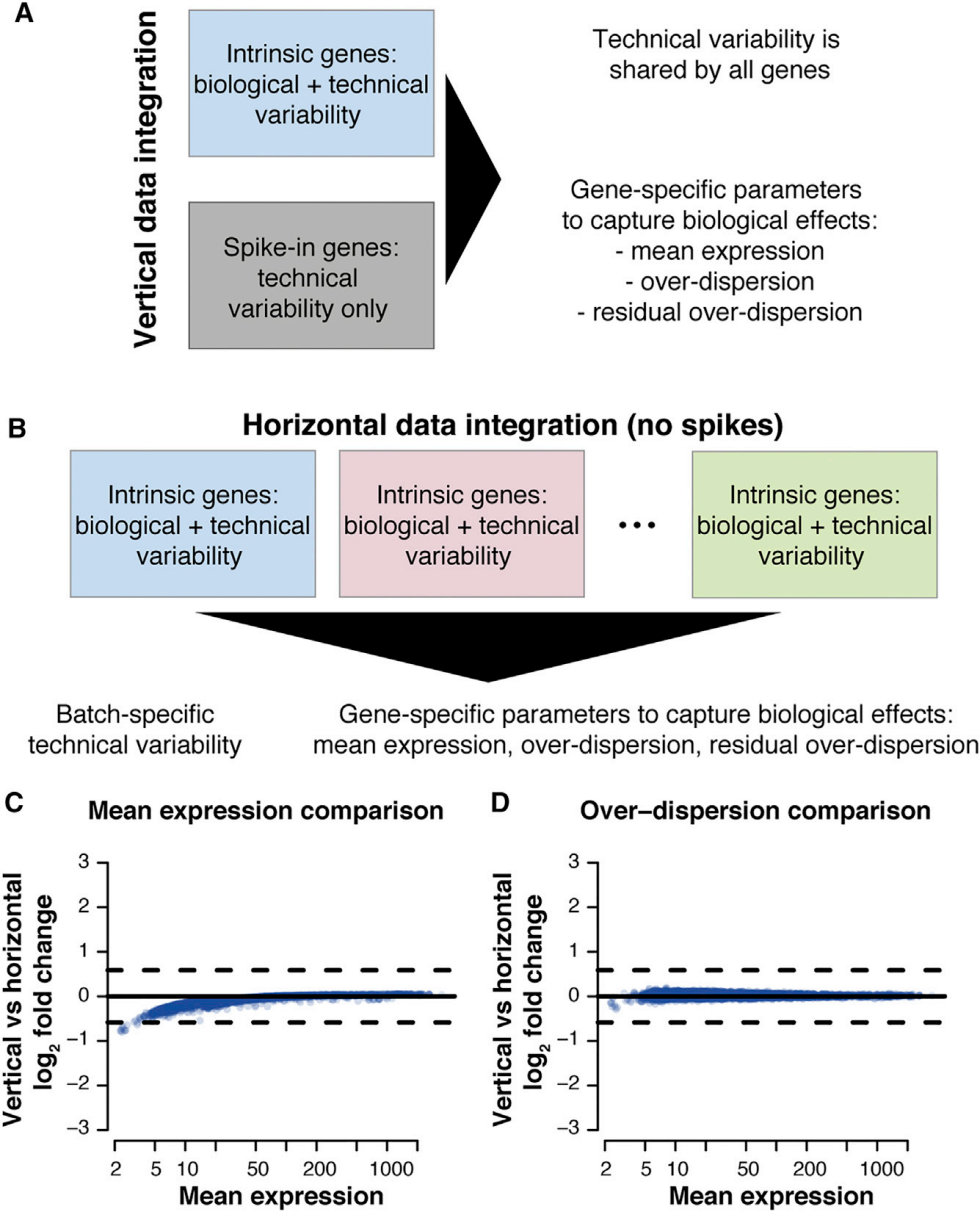
The Spikes and No-Spikes Implementations of BASiCS (A) Diagram representing the spikes implementation of BASiCS (Vallejos et al., 2015, 2016). This uses a vertical data integration approach to borrow information from gold-standard spike-in genes to aid normalization and to quantify technical variability. (B) Diagram representing the no-spikes implementation of BASiCS. This uses a horizontal data integration approach to borrow information across multiple batches of sequenced cells (not confounded by the biological effect of interest) to quantify technical variability. More details about this implementation are discussed in STAR Methods and Figure S4. (C and D) Comparison between the vertical and horizontal implementations of BASiCS using a dataset of mouse embryonic stem cells grown in 2i medium (see STAR Methods and Grün et al., 2014). Dashed horizontal lines located at ± log_2_(1.5) indicate the default minimum tolerance log_2_-fold change threshold used for differential testing. (C) Comparison in terms of posterior estimates for mean expression parameters *μ*_*i*_ across all genes. (D) Comparison in terms of posterior estimates for over-dispersion parameters *δ*_*i*_ across all genes. See also Figure S4.

Another critical aspect to take into account when inferring transcriptional variability based on scRNA-seq datasets is technical variation (Brennecke et al., 2013). BASiCS achieves this through a vertical data integration approach, exploiting a set of synthetic RNA spike-in molecules (e.g., the set of 92 ERCC molecules developed by Jiang et al. [2011]) as a *gold standard* to aid normalization and to quantify technical artefacts (see Figure 4A). However, while the addition of spike-in genes prior to sequencing is theoretically appealing (Lun et al., 2017), several practical limitations can preclude their utility in practice (Vallejos et al., 2017). Furthermore, the use of spike-in genes is not compatible with (increasingly popular) droplet-based technologies, which have massively increased the throughput of scRNA-seq over the last few years (Svensson et al., 2018).

Consequently, to ensure the broad applicability of our method, we extend BASiCS (both the regression and non-regression models) to handle datasets without spike-in genes. For this purpose, we exploit principles of measurement error models where—in the absence of gold standard features—technical variation is quantified through *replication* (Carroll, 1998). As described in Figure 4B, this horizontal data integration approach is based on experimental designs where cells from a population are randomly allocated to multiple independent experimental replicates (here referred to as “batches”). In such an experimental design, the no-spikes implementation of BASiCS assumes that biological effects are shared across batches and that technical variation will be reflected by spurious differences. As shown in Figures 4C and 4D, posterior inference under the no-spikes BASiCS model closely matches the original implementation for datasets where spike-ins and batches are available. Technical details about the no-spikes implementation of BASiCS are discussed in STAR Methods and Figure S4.

### Expression Variability Dynamics during Immune Activation and Differentiation

Here, we illustrate how our method assesses changes in expression variability using CD4^+^ T cells as a model system. For all datasets, pre-processing steps are described in STAR Methods.

### Testing Variability Changes in Immune Response Gene Expression

To identify gene expression changes during early T cell activation, we compared CD4^+^ T cells before (naive) and after (active) 3 hr of stimulation (Martinez-Jimenez et al., 2017). When using the non-regression BASiCS model, our differential over-dispersion test avoided the confounding with mean expression by solely focusing on genes with no changes in mean expression. This represents only a small fraction out of the full set of expressed genes. In contrast, testing changes in variability using residual over-dispersion measures allows testing across all genes, including the large set of genes that are up-regulated upon immune activation (see Figures S5A and S5B and STAR Methods). The latter include immune-response genes and critical drivers for CD4^+^ T cell functionality.

**Figure 5:**
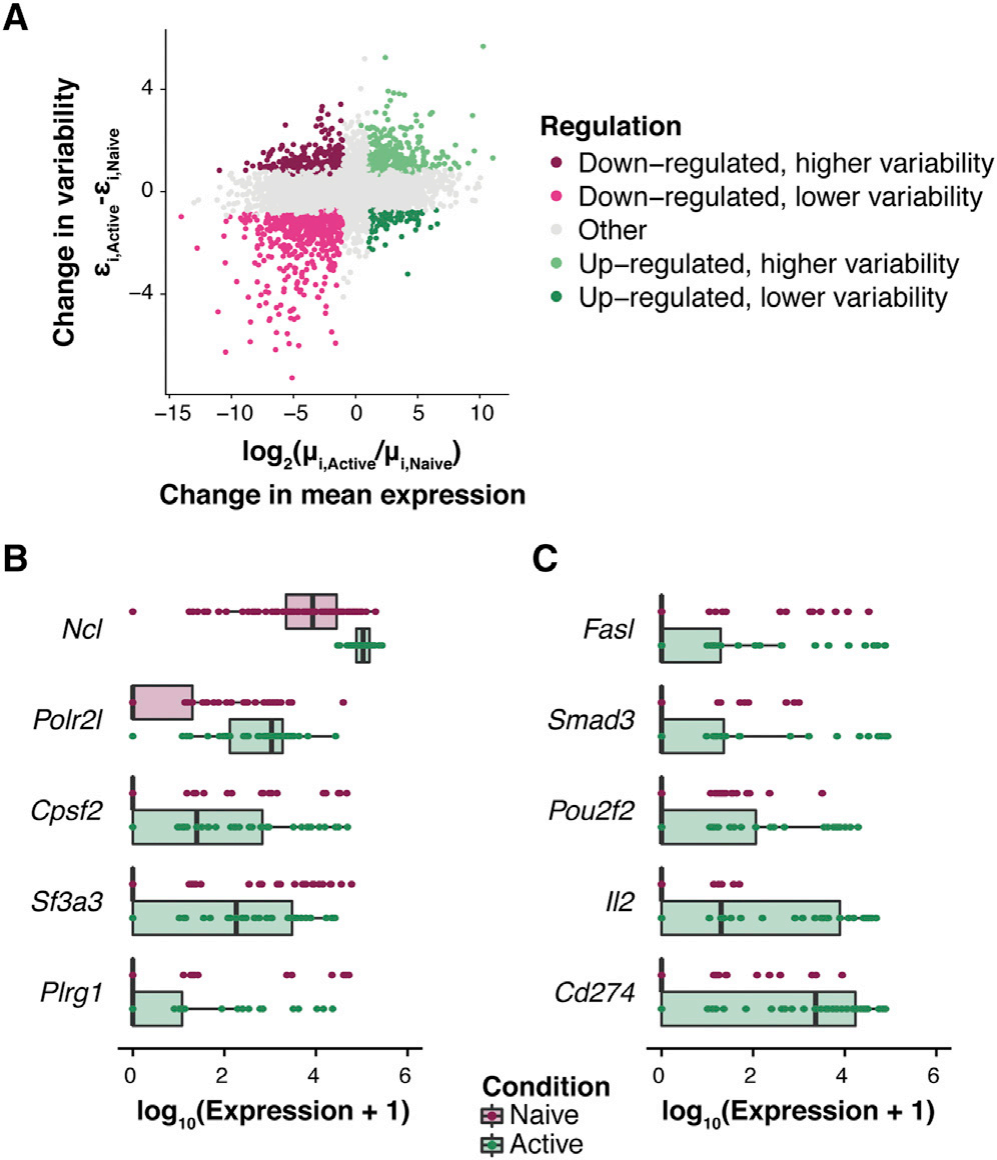
Changes in Expression Patterns during Early Immune Activation in CD4^+^ T Cells Differential testing (mean and residual over-dispersion) was performed between naive and activated murine CD4^+^ T cells. This analysis uses a minimum tolerance threshold of *τ*_0_ = 1 for changes in mean expression and a minimum tolerance threshold of *ψ*_0_ = 0.41 for differential residual over-dispersion testing (expected false discovery rate is fixed at 10%; see STAR Methods). (A) For each gene, the difference in residual over-dispersion estimates (Active versus Naive) is plotted versus the log_2_-fold change in mean expression (Active versus Naive). Genes with statistically significant changes in mean expression and variability are colored based on their regulation (up- or down-regulated, higher or lower variability). (B and C) Denoised expression counts across the naive (purple) and active (green) CD4^+^ T cell population are visualized for representative genes that (B) increase in mean expression and decrease in expression variability and (C) increase in mean expression as well as expression variability upon immune activation. Each dot represents a single cell. See also Figure S5.

Our model classifies genes into four categories based on their expression dynamics: down-regulated upon activation with (1) lower and (2) higher variability; and up-regulated with (3) lower and (4) higher variability (Figure 5A; STAR Methods; Table S2).

Genes with up-regulated expression upon activation and decreased expression variability encode components of the splicing machinery (e.g., *Sf3a3*, *Plrg1*), RNA polymerase subunits (e.g., *Polr2l*, *Polr1d*) as well as translation machinery components (e.g., *Ncl*, *Naf1*) (see Figure 5B). These biosynthetic processes help naive T cells to rapidly enter a program of proliferation and effector molecule synthesis (Tan et al., 2017, Araki et al., 2017). Therefore, rapid, uniform up-regulation of these transcripts would assist such processes. This observation also confirms previous findings that the translational machinery is tightly regulated during early immune activation (Martinez-Jimenez et al., 2017).

In contrast, genes with up-regulated expression and increased expression variability (see Figure 5C) include the death-inducing and inhibitory transmembrane ligands Fas ligand (*Fasl*) and PD-L1 (*Cd274*), the regulatory transcription factor Smad3 (*Smad3*), and the T cell receptor (TCR)-induced transcription factor, Oct2 (*Pou2f2*). Additionally, we detect a heterogeneous up-regulation in the mRNA expression of the autocrine and paracrine growth factor IL-2 (*Il2*) upon immune activation. This is in line with previous reports of binary IL-2 expression within a population of activated T cells, which has been suggested to be necessary for a scalable antigen response (Fuhrmann et al., 2016). Heterogeneity in expression of these genes suggests that despite the uniform up-regulation of biosynthetic machinery, T cells in this early activation state represent a mixed population with varying degrees of activation and/or regulatory potential.

We observe that for some genes (e.g., *Plrg1*), changes in variability are driven by a small number of outlier cells with high expression. The interpretation of these results is not trivial as it could reflect very subtle sub-structure or genuine changes in variability. To explore this, we performed the following synthetic experiment. We artificially created a mixed population of cells by combining 5 activated CD4^+^ T cells with a population of 93 naive CD4^+^ T cells (see STAR Methods). Subsequently, we performed a differential testing (mean and residual over-dispersion) between this mixed population and a *pure* population of 93 naive CD4^+^ T cells. As expected, this analysis shows an overall increase in variability in the mixed population. For example, among the genes that exhibit higher mean expression and higher residual over-dispersion in the mixed population, we found *Il2*—which is up-regulated upon CD4^+^ T cell activation (see Figure S5C). Moreover, we observe that the genes in this category are enriched for those that are only expressed in the 5 activated CD4^+^ T cells (see Figure S5D). This result suggests that differential variability testing can potentially uncover markers for heterogeneous cell states or cell types and can therefore provide important biological insights. However, changes in residual over-dispersion that are driven by outliers can also reflect unwanted contamination (e.g., mixed cell types), hence careful data filtering and clustering analysis should be performed prior to differential variability testing.

In summary, our approach allows us to extend the finding by Martinez-Jimenez et al. (2017), dissecting immune-response genes into two functional sets: (1) homogeneous up-regulation of biosynthetic machinery components and (2) heterogeneous up-regulation of several immunoregulatory genes.

### Expression Dynamics during *In Vivo* CD4^+^ T Cell Differentiation

In contrast to the quick transcriptional switch that occurs within hours of naive T cell activation, transcriptional changes during cellular differentiation processes are more subtle and were found to be coupled with changes in variability prior to cell fate decisions (Richard et al., 2016, Mojtahedi et al., 2016). Here, we apply our method to study changes in expression variability during CD4^+^ T cell differentiation after malaria infection using the dataset introduced by Lönnberg et al. (2017). In particular, we focus on samples collected 2, 4, and 7 days post malaria infection, for which more than 50 cells are available.

**Figure 6:**
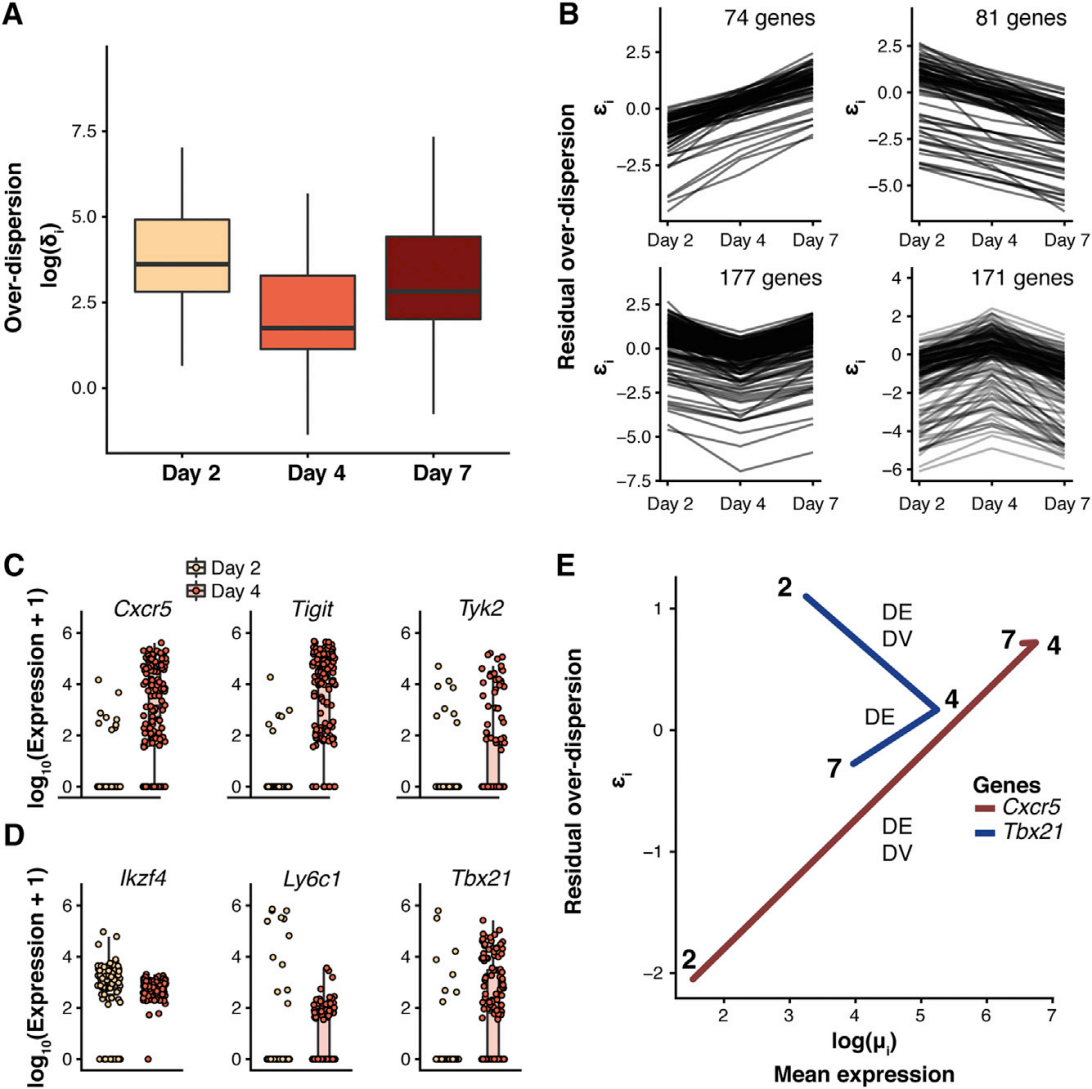
Dynamics of Expression Variability throughout CD4^+^ T Cell Differentiation Analysis was performed on CD4^+^ T cells assayed 2, 4, and 7 days after *Plasmodium* infection. Changes in residual over-dispersion were tested using a minimum tolerance threshold of *ψ*_0_ = 0.41 (expected false discovery rate is fixed at 10%; see STAR Methods) (A) Distribution of posterior estimates of over-dispersion parameters *δ*_*i*_ for genes that exhibit no changes in mean expression across the differentiation time course. Changes in mean expression were tested using a minimum tolerance threshold of *τ*_0_ = 0 (expected false discovery rate is fixed at 10%). (B) Posterior estimates for residual over-dispersion parameters $\epsilon_{i}$, focusing on genes with statistically significant changes in expression variability between time points. Gene set size is indicated for each plot. (C and D) Denoised expression counts across cell populations at days 2 (yellow) and 4 (red) post infection are visualized for representative genes that (C) increase or (D) decrease in variability during differentiation. Each dot represents a single cell. (E) *Tbx21* (blue) and *Cxcr5* (red) measured at days 2, 4, and 7 post infection. Posterior estimates for residual over-dispersion parameters $\epsilon_{i}$ are plotted against posterior estimates for mean expression parameters *μ*_*i*_. Statistically significant changes in mean expression (DE, minimum tolerance threshold of *τ*_0_ = 1) and variability (DV, minimum tolerance threshold of *ψ*_0_ = 0:41) are indicated for each comparison (expected false discovery rate is fixed at 10%). See also Figure S6.

To study global changes in over-dispersion along the differentiation time course, we first compared posterior estimates for the gene-specific parameter *δ*_*i*_, focusing on genes for which mean expression does not change (see Figure 6A and STAR Methods). This analysis suggests that the expression of these genes is most tightly regulated at day 4, when cells are in a highly proliferative state. Moreover, between days 4 and 7, the cell population becomes more heterogeneous. This is in line with the emergence of differentiated T helper (Th) 1 and Tfh cells that was observed by Lönnberg et al. (2017).

We next exploited the residual over-dispersion parameters to identify changes in variability (irrespective of changes in mean expression) between consecutive time points (see STAR Methods). For example, separating these genes by whether their variability increases or decreases between time points revealed four different patterns (see Figure 6B). These include genes whose variability systematically increases (or decreases) as well as patterns where variability is highest (or lowest) at day 4.

In particular, differential variability analysis between days 2 and 4 revealed changes in expression variability for a set of immune-related genes (see Figure 6C). For example, expression of *Cxcr5*, which encodes the chemokine receptor that directs Tfh cells to the B cell follicles (Crotty, 2014), strongly increases in variability on day 4. This finding agrees with results from Lönnberg et al. (2017), where Tfh and Th1 differentiation was observed to be transcriptionally detectable at day 4 within a subset of activated cells. A similar behavior was observed for *Tyk2* and *Tigit*. The latter encodes a receptor that is expressed by a subset of Tfh cells and was found to promote Tfh function (Godefroy et al., 2015). In contrast, we observed a decrease in variability between days 2 and 4 for *Ikzf4* (Treg-associated gene), *Ly6c1* (expressed by effector T cells), and *Tbx21* (encoding the Th1 lineage-defining transcription factor Tbet). Subsequently, we summarized the results of our differential testing between days 2 and 4 as well as days 4 and 7, focusing on genes that were previously detected to be Th1- or Tfh-lineage associated (Lönnberg et al., 2017). We detected a continuous increase in expression of Th1-associated genes but not Tfh-associated genes (see Figure S6A and STAR Methods), with the majority of changes in variability for these genes occurring between days 2 and 4.

We next examined immune-related genes (*Il2ra, Tbx21, Il2rb, Cxcr5, Selplg, Id2, Ifng, Icos, Ifngr1*) that were previously described as showing differences in their peak expression over the pseudotime course of differentiation (Figure S6B; STAR Methods; Lönnberg et al., 2017). From this list, the lineage-associated genes *Tbx21* and *Cxcr5* are up-regulated between days 2 and 4. However, these genes exhibit opposite behaviors in terms of variability: *Cxcr5* increases and *Tbx21* decreases in variability between days 2 and 4 (see Figure 6D). The fact that variability of *Tbx21* (Tbet) expression was highest on day 2 suggests that Tbet is up-regulated very early in differentiation, as seen in Lönnberg et al. (2017) and similar to *in vitro* Th1 induction (Szabo et al., 2000). Moreover, this suggests that Th1 fate decisions (for at least a subset of cells) may be made even earlier than the differentiation bifurcation point identified on day 4 by the original study (Lönnberg et al., 2017).

## Discussion

In recent years, the importance of modulating cell-to-cell transcriptional variation within cell populations for tissue function maintenance and development has become apparent (Bahar Halpern et al., 2015, Mojtahedi et al., 2016, Goolam et al., 2016). Here, we present a statistical approach to robustly test changes in expression variability between cell populations using scRNA-seq data. Our method uses a hierarchical Bayes formulation to extend the BASiCS framework by addressing (increasingly popular) experimental protocols where spike-in sequences are not available and by incorporating an additional set of residual over-dispersion parameters $\epsilon_{i}$ that are not confounded by changes in mean expression. Together, these extensions ensure a broader applicability of the BASiCS software and allow statistical testing of changes in variability that are not confounded by technical noise or mean expression.

In general, stable gene-specific variability estimates ideally require a large and deeply sequenced dataset containing a homogeneous cell population (the use of unique molecular identifiers for quantifying transcript counts can also improve variability estimation; see Grün et al. [2014]). However, we observe that the regression BASiCS model leads to a more stable inference that requires fewer cells to accurately estimate gene-specific summaries, particularly for lowly expressed genes. Despite this, careful considerations should be taken in extreme scenarios where the number of cells is small and/or the data are highly sparse (e.g., droplet-based approaches). These features of the data not only affect parameter estimation but also downstream differential testing. For sparse datasets with low numbers of cells, we recommend the use of a stringent minimum tolerance threshold and/or calibrating the test to a low expected false discovery rate (e.g., 1%) to avoid detecting spurious signals. Moreover, if possible, an internal calibration can be performed to find a reasonable minimum tolerance threshold (e.g., by randomly permuting cells between two groups to calibrate the null distribution of the differences between populations).

Our method allows characterization of the extent and nature of variable gene expression in CD4^+^ T cell activation and differentiation. First, we observe that during acute activation of naive T cells, genes of the biosynthetic machinery are homogeneously up-regulated, while specific immune-related genes become more heterogeneously up-regulated. In particular, increased variability in expression of the apoptosis-inducing Fas ligand (Strasser et al., 2009) and the inhibitory ligand PD-L1 (Chikuma, 2016) suggests a mechanism by which newly activated cells might suppress re-activation of effector cells, thereby dynamically modulating the population response to activation. Likewise, more variable expression of Smad3, which translates inhibitory TGF*β* signals into transcriptional changes (Delisle et al., 2013), may indicate increased diversity in cellular responses to this signal. Increased variability in *Pou2f2* (Oct2) expression after activation suggests heterogeneous activities of the NF-*κ*B and/or NFAT signaling cascades that control its expression (Mueller et al., 2013). Moreover, we detect up-regulated and more variable *Il2* expression, suggesting heterogeneous IL-2 protein expression, which is known to enable T cell population responses (Fuhrmann et al., 2016).

Finally, we studied changes in gene expression variability during CD4^+^ T cell differentiation toward a Th1 and Tfh cell state over a 7-day time course after *in-vivo* malaria infection (Lönnberg et al., 2017). Our analysis provides several insights into this differentiation system. First, we observe a tighter regulation in gene expression among genes that do not change in mean expression during differentiation at day 4, when divergence of Th1 and Tfh differentiation was previously identified (Lönnberg et al., 2017). This decrease in variability on day 4 is potentially due to the induction of a strong pan-lineage proliferation program. However, we observe that not all genes follow this trend and uncover four different patterns of variability changes. Second, we observe that several Tfh and Th1 lineage-associated genes change in expression variability between days 2 and 4. For example, we noted a decrease in variability for one key Th1 regulator, *Tbx21* (encoding Tbet), which suggests that a subset of cells may have already committed to the Th1 lineage at day 2. Three additional Th1 lineage-associated genes also followed this trend (*Ahnak*, *Ctsd*, *Tmem154*). These data suggest that differentiation fate decisions may arise as early as day 2 in subpopulations within this system, resulting in high gene expression variability. Such an effect is in accordance with the early commitment to effector T cell fates that was previously observed during viral infection (Choi et al., 2011). As these results illustrate, diversity in differentiation state within a population of T cells can drive our differential variability results. To further dissect these results, subsequent analyses such as the pseudotime inference used in Lönnberg et al. (2017) could be used to characterize a continuous differentiation process.

In sum, our model provides a robust tool for understanding the role of heterogeneity in gene expression during cell fate decisions. With the increasing use of scRNA-seq to study this phenomenon, ours and other related tools will become increasingly important.

## STAR★Methods

### Key Resources Table

**Table:** 

REAGENT or RESOURCE	SOURCE	IDENTIFIER
**Deposited Data**		
Mouse genome reference GRCm38	ENSEMBL	ftp://ftp.ensembl.org/pub/release-91/fasta/mus_musculus/dna/
Naive and activated CD4+ T cells	Martinez-Jimenez et al. (2017)	Array Express: E-MTAB-4888
Differentiating CD4+ T cells	Lönnberg et al. (2017)	Array Express: E-MTAB-4388
Mouse brain cells	Zeisel et al. (2015)	GEO: GSE60361
*Dictyostelium* cells	Antolović et al. (2017)	Data S1 in original publication
Pool-split RNA	Grün et al. (2014)	GEO: GSE54695
**Software and Algorithms**		
BASiCS version 1.1.57	This paper	https://www.bioconductor.org/packages/3.7/bioc/html/BASiCS.html
Gsnap v2014-12-29	Wu and Nacu (2010)	https://github.com/juliangehring/GMAP-GSNAP
HTSeq v0.6	Anders et al. (2015)	https://htseq.readthedocs.io
DAVID v6.8	Dennis et al. (2003)	https://david.ncifcrf.gov/

### Contact for Reagent and Resource Sharing

Further information and requests for resources and reagents should be directed to and will be fulfilled by the Lead Contact, John Marioni (john.marioni@cruk.cam.ac.uk).

### Method Details

#### The BASiCS Framework

The proposed statistical model builds upon BASiCS (Vallejos et al., 2015, Vallejos et al., 2016) — an integrated Bayesian framework that infers technical noise in scRNA-seq datasets and simultaneously performs data normalisation as well as selected supervised downstream analyses.

Let *X*_*ij*_ be a random variable representing the expression count of gene *i* (∈{1, …, *q*}) in cell *j* (∈{1, …, *n*}). To control for technical noise, we employ reads from synthetic RNA spike-ins (e.g. those introduced by Jiang et al., 2011). Without loss of generality, we assume the first *q*_0_ genes to be biological followed by the *q* − *q*_0_ spike-in genes. As in the original BASiCS method introduced by Vallejos et al. (2015), we assume a Poisson hierarchical formulation:

(Equation 1) $$X_{ij}|\mu_{i},\phi_{j},\nu_{j},\rho_{ij}\overset{\text{ind}}{∼}\{\begin{array}{ll} \text{Poisson}\left(\phi_{j}\nu_{j}\mu_{i}\rho_{ij}\right), & i=1,...,q_{0},j=1,...n;\\ \text{Poisson}\left(\nu_{j}\mu_{i}\right), & i=q_{0}+1,...,q,j=1,...,n, \end{array}$$

where, to account for technical and biological factors that affect the variance of the transcript counts, we incorporate two random effects:

(Equation 2) $$\nu_{j}|s_{j},\theta \overset{\text{ind}}{∼}\text{Gamma}\;\left(\frac{1}{\theta },\frac{1}{s_{j}\theta }\right)\text{,}\;\rho_{ij}|\delta_{i}\overset{\text{ind}}{∼}\text{Gamma}\;\left(\frac{1}{\delta_{i}},\frac{1}{\delta_{i}}\right).$$

In this setup, *Φ*_*j*_ represents a cell-specific normalization parameter to correct for differences in mRNA content between cells and *s*_*j*_ models cell-specific scale differences affecting all biological and technical genes. Moreover, the random effect *v*_*j*_ captures unexplained technical noise that is not accounted for by the normalisation. The strength of this noise is then quantified by a global parameter θ (shared across all genes and cells). Heterogeneous gene expression across cells is captured by *ρ*_*ij*_, whose strength is controlled by gene-specific over-dispersion parameters *δ*_*i*_. These quantify the excess of variability that is observed with respect to Poisson sampling noise, after accounting for technical noise. Finally, gene-specific parameters $\mu_{i}$ represent average expression of a gene across cells.

When comparing two or more groups of cells (e.g. experimental conditions or cell types), the notation above can be extended by assuming that gene-specific parameters are also group-specific (as in Vallejos et al., 2016). Comparisons of gene-specific parameters across populations can be used to identify statistically significant changes in gene expression at the mean and the variability level. However, the well known confounding effect between mean and variability that typically arises in scRNA-seq datasets (Brennecke et al., 2013) can preclude a meaningful interpretation of these results.

#### Modeling the Confounding between Mean and Dispersion

Here, we extend BASiCS to account for the confounding effect described above. For this purpose, we estimate the relationship between mean and over-dispersion parameters by introducing the following joint prior distribution for ${\left(\mu_{i},\delta_{i}\right)}^{\prime }$:

(Equation 3) $$\mu_{i}∼\text{log}\text{-Normal}\left(0,s_{\mu }^{2}\right),\;\delta_{i}|\mu_{i}∼\text{log}\text{-}t_{\eta }\left(\text{f}\left(\mu_{i}\right),\sigma^{2}\right).$$

The latter is equivalent to the following non-linear regression model:

(Equation 4) $$\log\left(\delta_{i}\right)=\text{f}\left(\mu_{i}\right)+\epsilon_{i},\;\epsilon_{i}∼t_{\eta }\left(0,\sigma^{2}\right),$$

where $\text{f}\left(\mu_{i}\right)$ represents the over-dispersion (on the log-scale) that is predicted by the global trend (across all genes) expressed at a given mean expression $\mu_{i}$. Therefore, $\epsilon_{i}$ can be interpreted as a latent gene-specific *residual over-dispersion* parameter, capturing departures from the overall trend. If a gene exhibits a positive value for $\epsilon_{i}$, this indicates more variation than expected for genes with similar expression level. Accordingly, negative values of $\epsilon_{i}$ suggest less variation than expected for genes with similar expression level.

A similar approach was introduced by DESeq2 (Love et al., 2014) in the context of bulk RNA sequencing. Whereas DESeq2 assumes normally distributed errors when estimating this trend, here we opt for a Student-*t* distribution as it leads to inference that is more robust to the presence of outlier genes. Moreover, the parametric trend assumed by DESeq2 is replaced by a more flexible semi-parametric approach. This is defined by

(Equation 5) $$f\left(\mu_{i}\right)=\alpha_{0}+\log\left(\mu_{i}\right)\alpha_{1}+\sum_{l=1}^{L}g_{l}\left(\log\left(\mu_{i}\right)\right)\beta_{l},$$

where *g*_1_(⋅),…,*g*_*L*_(⋅) represent a set of Gaussian radial basis function (GRBF) kernels and $\alpha_{0},\alpha_{1},\beta_{1},\ldots ,\beta_{L}$ are regression coefficients. As in Kapourani and Sanguinetti (2016), the GRBF kernels are defined as:

(Equation 6) $$g_{l}\left(\log\left(\mu_{i}\right)\right)=\;\exp\left\{-\frac{1}{2}{\left(\frac{\log\left(\mu_{i}\right)-m_{l}}{h_{l}}\right)}^{2}\right\},\;l=1,\ldots ,L,$$

where *m_l_* and *h*_*l*_ represent location and scale hyper-parameters for GRBF kernels.

In Equation 5, the linear term captures the (typically negative) global correlation between $\delta_{i}$ and $\mu_{i}$. Its addition also stabilises inference of GRBFs around mean expression values where only a handful of genes are observed. In Equation 6, the location and scale hyper-parameters (*m*_*l*_, *h*_*l*_) are assumed to be fixed *a priori*. Details about this choice are described below.

The remaining elements of the prior were chosen as follows:

(Equation 7) $$\beta |\sigma^{2}∼\text{Normal}\left(m_{\beta },\sigma^{2}V_{\beta }\right),$$

(Equation 8) $$\sigma^{2}∼\text{Inv}\text{-}\text{Gamma}\left(a_{\sigma^{2}},b_{\sigma^{2}}\right),$$

(Equation 9) $$s_{j}\overset{\text{iid}}{∼}\text{Gamma}\left(a_{s},b_{s}\right),\;j=1,\ldots n,$$

(Equation 10) $${\left(\phi_{1},\ldots ,\phi_{n}\right)}^{\prime }∼n\times \text{Dirichlet}\left(a_{\phi }\right),$$

(Equation 11) $$\theta ∼\text{Gamma}\left(a_{\theta },b_{\theta }\right),$$

with all hyper-parameters fixed *a priori*. Default values are chosen as:

(Equation 12) $$m_{\beta }=0_{L}\left(\text{an}\;L\text{-}\text{dimensional}\;\text{vector}\;\text{of}\;\text{zeroes}\right),$$

(Equation 13) $$V_{\beta }=I_{L}\;\left(\text{an}\;L\text{-}\text{dimensional}\;\text{identity}\;\text{matrix}\right),$$

(Equation 14) $$a_{\sigma^{2}}=2,$$

(Equation 15) $$a_{\sigma^{2}}=2,$$

with the remaining default hyper-parameter values as in Vallejos et al. (2016).

In principle, the degrees of freedom parameter η could also be estimated within a Bayesian framework. However, we observed that fixing this parameter *a priori* led to more stable results. A default choice for this parameter is described below.

#### Implementation

Posterior inference for the model described above is implemented by extending the Adaptive Metropolis within Gibbs sampler (Roberts and Rosenthal, 2009) that was adopted by Vallejos et al. (2016). For this purpose, the log-Student-*t* distribution in Equation 3 is represented via the same data augmentation scheme as in Vallejos and Steel (2015). The latter introduces an auxiliary set of parameters $\lambda_{i}$ such that:

(Equation 16) $$\delta_{i}|\mu_{i},\beta ,\sigma^{2},\lambda_{i},\eta \overset{\text{ind}}{∼}\text{log}\text{-}\text{Normal}\;\left(f\left(\mu_{i}\right),\frac{\sigma^{2}}{\lambda_{i}}\right),\;\lambda_{i}|\eta \overset{\text{ind}}{∼}\text{Gamma}\left(\frac{\eta }{2},\frac{\eta }{2}\right).$$

Moreover, the regression coefficients $\beta ={\left(\alpha_{0},\alpha_{1},\beta_{1},\ldots ,\beta_{L}\right)}^{\prime }$ are inferred by noting that Equation 5 can be rewritten as a linear regression model using

(Equation 17) $$f\left(\mu_{i}\right)=X\beta ,$$

where *X* is a *q*_0_×(L+2) matrix given by

(Equation 18) $$X=\left(\begin{array}{ccccc} 1 & \log\left(\mu_{1}\right) & g_{1}\left(\log\left(\mu_{1}\right)\right) & \cdots & g_{L}\left(\log\left(\mu_{1}\right)\right)\\ 1 & \log\left(\mu_{2}\right) & g_{1}\left(\log\left(\mu_{2}\right)\right) & \cdots & g_{L}\left(\log\left(\mu_{2}\right)\right)\\ \vdots & \vdots & \vdots & \ddots & \vdots \\ 1 & \log\left(\mu_{q_{0}}\right) & g_{1}\left(\log\left(\mu_{q_{0}}\right)\right) & \cdots & g_{L}\left(\log\left(\mu_{q_{0}}\right)\right) \end{array}\right).$$

In this setting, the full conditionals associated with *s*_*j*_, φ_*j*_, *v*_*j*_ and *θ* are not affected by the new prior specification of ${\left(\mu_{i},\delta_{i}\right)}^{\prime }$ and can be found in Vallejos et al. (2016). The full conditionals for $\mu_{i}$, $\delta_{i}$, β, and $\sigma^{2}$ are derived below. As in Vallejos et al. (2015), these are derived by integrating out the random effect $\rho_{ij}$ in Equation 1, leading to:

(Equation 19) $$X_{ij}|\mu_{i},\delta_{i},\phi_{j},\nu_{j}\overset{\text{ind}}{∼}\left\{ \begin{array}{ll} \text{Neg}\text{-}\text{Bin}\;\left(\frac{1}{\delta_{i}},\frac{\phi_{j}\nu_{j}\mu_{i}}{\phi_{j}\nu_{j}\mu_{i}+\frac{1}{\delta_{i}}}\right), & i=1,...,q_{0},j=1,...n;\\ \text{Poisson}\left(\nu_{j}\mu_{i}\right), & i=q_{0}+1,...,q,j=1,...,n \end{array}\right.$$

Based on Equation 19, the likelihood function therefore takes the form

$$\left[\prod_{i=1}^{q_{0}}\prod_{j=1}^{n}\frac{\Gamma \left(x_{ij}+\frac{1}{\delta_{i}}\right)}{\Gamma \left(\frac{1}{\delta_{i}}\right)x_{ij}!}{\left(\frac{\frac{1}{\delta_{i}}}{\phi_{j}\nu_{j}\mu_{i}+\frac{1}{\delta_{i}}}\right)}^{\frac{1}{\delta_{i}}}{\left(\frac{\phi_{j}\nu_{j}\mu_{i}}{\phi_{j}\nu_{j}\mu_{i}+\frac{1}{\delta_{i}}}\right)}^{x_{ij}}\right]$$

(Equation 20) $$\times \left[\prod_{i=q_{0}+1}^{q}\prod_{j=1}^{n}\frac{{\left(\nu_{j}\mu_{i}\right)}^{x_{ij}}}{x_{ij}!}\exp\left\{-\nu_{j}\mu_{i}\right\}\right]\times \left[\prod_{j=1}^{n}\frac{{\left(s_{j}\theta \right)}^{-\frac{1}{\theta }}}{\Gamma \left(\frac{1}{\theta }\right)}\nu_{j}^{\frac{1}{\theta }-1}\;\exp\left\{-\frac{\nu_{j}}{s_{j}\theta }\right\}\right].$$

Let $f\left(\mu_{i}\right)$ be as in Equation 5. The full conditionals associated to the mean expression parameters $\mu_{i}$ and over-dispersion parameters $\delta_{i}$ are respectively given by:

(Equation 21) $$\pi \left(\mu_{i}|\cdot \right)\propto \frac{\mu_{i}^{\sum_{j=1}^{n}x_{ij}}}{\prod_{j=1}^{n}{\left(\phi_{j}\nu_{j}\mu_{i}+\frac{1}{\delta_{i}}\right)}^{\frac{1}{\delta_{i}}+x_{ij}}}\exp\left\{-\frac{{\left(\log\left(\mu_{i}\right)\right)}^{2}}{2a_{\mu }^{2}}-\frac{{\left(\log\left(\delta_{i}\right)-f\left(\mu_{i}\right)\right)}^{2}}{2\sigma^{2}/\lambda_{i}}\right\}\frac{1}{\mu_{i}},$$

(Equation 22) $$\pi \left(\delta_{i}|\cdot \right)\propto \left[\prod_{j=1}^{n}\frac{\Gamma \left(x_{ij}+\frac{1}{\delta_{i}}\right)}{\Gamma \left(\frac{1}{\delta_{i}}\right)}\frac{{\left(\frac{1}{\delta_{i}}\right)}^{\frac{1}{\delta_{i}}}}{{\left(\phi_{j}\nu_{j}\mu_{i}+\frac{1}{\delta_{i}}\right)}^{\frac{1}{\delta_{i}}+x_{ij}}}\right]\exp\left\{-\frac{{\left(\log\left(\delta_{i}\right)-f\left(\mu_{i}\right)\right)}^{2}}{2\sigma^{2}/\lambda_{i}}\right\}\frac{1}{\delta_{i}}.$$

Moreover, the full conditionals associated to the remaining parameters $\lambda_{i}$, β and $\sigma^{2}$ are given by

(Equation 23) $$\lambda_{i}|\cdot \overset{\text{ind}}{∼}\text{Gamma}\left(a_{\lambda_{i}}^{*},b_{\lambda_{i}}^{*}\right),\;i=1,\ldots ,q_{0},$$

(Equation 24) $$\beta |\cdot ∼N\left(m_{\beta }^{*},\sigma^{2}V_{\beta }^{*}\right),$$

(Equation 25) $$\sigma^{2}|\cdot ∼\text{Inv}\text{-}\text{Gamma}\left(a_{\sigma^{2}}^{*},b_{\sigma^{2}}^{*}\right),$$

with

(Equation 26) $$a_{\sigma^{2}}=2,$$

(Equation 27) $$a_{\sigma^{2}}=2,$$

(Equation 28) $$a_{\sigma^{2}}=2,$$

(Equation 29) $$m_{\beta }^{*}={\left(X^{\prime }\Lambda X+V_{\beta }^{-1}\right)}^{-1}\left(X^{\prime }\Lambda Y+V_{\beta }^{-1}m_{\beta }\right),$$

(Equation 30) $$a_{\sigma^{2}}=2,$$

(Equation 31) $$b_{\sigma^{2}}^{*}=b_{\sigma^{2}}+\frac{1}{2}\left(Y^{\prime }\Lambda Y+m_{\beta }^{\prime }V_{\beta }^{-1}m_{\beta }+{\left(\beta -m_{\beta }^{*}\right)}^{\prime }{\left(V_{\beta }^{*}\right)}^{-1}\left(\beta -m_{\beta }^{*}\right)-{\left(m_{\beta }^{*}\right)}^{\prime }{\left(V_{\beta }^{*}\right)}^{-1}m_{\beta }^{*}\right),$$

(Equation 32) $$\equiv b_{\sigma^{2}}+\frac{1}{2}\left(Y^{\prime }\Lambda Y+m_{\beta }^{\prime }V_{\beta }^{-1}m_{\beta }+\beta^{\prime }{\left(V_{\beta }^{*}\right)}^{-1}\beta -2\beta^{\prime }{\left(V_{\beta }^{*}\right)}^{-1}m_{\beta }^{*}\right),$$

where Λ is a diagonal matrix with elements $\left(\lambda_{1},\ldots ,\lambda_{q_{0}}\right)$ and $Y={\left(\log\left(\delta_{1}\right),\ldots ,\;\log\left(\delta_{q_{0}}\right)\right)}^{'}$. Finally, the full conditionals associated to the global technical noise parameter (*θ*) and cell-specific parameters (*Φ*_*j*_, *s*_*j*_ and *v*_*j*_) are defined as in Vallejos et al. (2016).

#### Probabilistic Rule Associated to the Differential Test

We use a probabilistic approach to identify changes in gene expression between groups of cells. Let $\delta_{i}^{A}$ and $\delta_{i}^{B}$ be the over-dispersion parameters associated to gene *i* in groups *A* and *B*. Following Equation 4, the log_2_ fold change in over-dispersion between these groups can be decomposed as:

(Equation 33) $$\log_{2}\;\left(\frac{\delta_{i}^{A}}{\delta_{i}^{B}}\right)=\log_{2}\left(e\right)\times \left[\underset{\text{Mean}\;\text{contribution}}{\underset{︸}{f^{A}\left(\mu_{i}^{A}\right)-f^{B}\left(\mu_{i}^{B}\right)}}+\underset{\text{Residual}\;\text{change}}{\underset{︸}{\epsilon_{i}^{A}-\epsilon_{i}^{B}}}\right],$$

where the first term captures the over-dispersion change that can be attributed to differences between $\mu_{i}^{A}$ and $\mu_{i}^{B}$. The second term in Equation 33 represents the change in residual over-dispersion that is not confounded by mean expression. Based on this observation, statistically significant differences in residual over-dispersion will be identified for those genes where the tail posterior probability of observing a large difference between $\epsilon_{i}^{A}$ and $\epsilon_{i}^{B}$ exceeds a certain threshold, i.e.

(Equation 34) $$\text{P}\left(\left|\epsilon_{i}^{A}-\epsilon_{i}^{B}\right|>\psi_{0}|\text{Data}\right)>\alpha_{R},$$

where $\psi_{0}>0$ defines a pre-specified minimum tolerance threshold. As a default choice, we assume $\psi_{0}=\log_{2}\left(1.5\right)/\log_{2}\left(e\right)\approx 0.41$ which translates into a 50% increase in over-dispersion. In the limiting case when $\psi_{0}=0$, the probability in Equation 34 is equal to 1 regardless of the information contained in the data. Therefore, as in Bochkina and Richardson (2007), our decision rule is based on the maximum of the posterior probabilities associated to the one-sided hypotheses ${\epsilon_{i}}^{A}-\epsilon_{i}^{B}>0$ and ${\epsilon_{i}}^{A}-\epsilon_{i}^{B}<0$, i.e.

(Equation 35) $$2\times \max\left\{\pi_{i},1-\pi_{i}\right\}-1>\alpha_{R},\;\text{with}\;\pi_{i}=\text{P}\left(\epsilon_{i}^{A}-\epsilon_{i}^{B}>0|\text{Data}\right).$$

In both cases, the posterior probability threshold $\alpha_{R}$ is chosen to control the expected false discovery rate (EFDR) (Newton et al., 2004). The default value for EFDR is set to 10%. As a default and to support interpretability of the results, we exclude genes that are not expressed in at least 2 cells per condition from differential variability testing.

Changes in mean and over-dispersion are highlighted using the decision rule of Vallejos et al. (2016).

To evaluate the performance of our differential test we generated synthetic data under a null model (without changes in variability) and an alternative model (with changes in variability). All datasets were generated following the BASiCS model, with parameter values used set by empirical estimates based on 98 microglia cells (see below). For this purpose, we use the *BASiCS_Sim* function. To simulate data under an alternative model, 1000 genes were randomly selected and their associated $\delta_{i}$’s were increased or decreased by a log_2_ fold change of 5. Differential testing was performed either between data simulated on the same set of parameters (null model) or between data simulated from the original parameters and the altered parameters (alternative model). We report the EFDR (Newton et al., 2004) as well as the false positive rate (FPR) for simulations under the null model and the true positive rate (TPR) for simulations under the alternative model. Synthetic data were generated with different sample sizes, with 5 repetitions for each sample size (see Figure S1)

#### Choice of Hyper-Parameters

As discussed above, the degrees of freedom η, the number of GRBFs *L* as well as the associated hyper-parameters (*m*_*l*_, *h*_*l*_) are set *a priori*. Here, we explain the default values implemented in the BASiCS software. These were chosen to achieve a compromise between flexibility and shrinkage strength when applied to the datasets described in Table S1.

Firstly, we observed that large values of *L* can lead to over-fitting but that small values of *L* can limit the flexibility to capture non-linear relations between $\log\left(\delta_{i}\right)$ and $\log\left(\mu_{i}\right)$. Thus, as a parsimonious choice, we selected *L*=10. Moreover, as in Kapourani and Sanguinetti (2016), values for *m_l_* were chosen to be equally spaced across the range of $\log\left(\mu_{i}\right)$, i.e.

(Equation 36) $$m_{l}=a+\left(l-1\right)\frac{b-a}{L-1},\;l=1,\ldots ,L,$$

where $a=\min_{i\in \left\{1,\ldots ,q_{0}\right\}}\left\{\log\left(\mu_{i}\right)\right\}$ and $b=\max_{i\in \left\{1,\ldots ,q_{0}\right\}}\left\{\log\left(\mu_{i}\right)\right\}$. As $\mu_{i}$ values are unknown *a priori*, *a* and *b* are updated every 50 MCMC iterations during burn-in (fixed thereafter). Additionally, the scale hyper-parameters *h*_*l*_ control the width of the GRBFs and, consequently, the locality of the regression. As a default, we set these as *h*_*l*_=*c*×Δ*m*, where *c* is a fixed proportionality constant and Δ*m* is the distance between consecutive values of *m_l_*. In practice, we observed that the choice of a particular value of *c* is not critical, as long as narrow kernels (*c*<0.5) are avoided. As a default, *c*=1.2 was chosen.

The degrees of freedom η controls the tails of the distribution for the residual term in Equation 4. This influences the shrinkage towards the global trend and the robustness against outlying observations (here, these refer to genes whose mean and over-dispersion values are far from the trend). If η≥30, $\epsilon_{i}$ approximately follows a normal distribution for which posterior inference for β is known to be sensitive to outliers. Instead, small values of η introduce heavy-tails for $\epsilon_{i}$, leading to more robust posterior inference. In principle, η could be estimated within a Bayesian framework. However, this is problematic as the likelihood function associated to Equation 4 can be unbounded (Fernandez and Steel, 1999). Here, we opt for a pragmatic approach where the value of η is fixed *a priori*. To select a reasonable default value, we ran the regression BASiCS model for a grid of possible values of η (η∈{1,2,3,4,5,6,7,8,9,15,20,25,30}), using the datasets described in Table S1 (with *L*, *m*_*l*_ and *h_l_* fixed as described above). In all cases, we calculated Monte Carlo estimates for the log-likelihood associated to Equation 1 as a proxy for goodness-of-fit (data not shown). We observed that log-likelihood estimates were consistently the smallest for η=1 and that no substantial differences are observed across larger values of η (provided that η<<30).

Based on these observations, default values implemented in the BASiCS software are set to *L*=10, *c*=1.2, η=5. Despite this, the model’s implementation also allows flexible adjustment of *L*, *c* and η by the user.

#### Running the Different Implementations of BASiCS

In the BASiCS R library, the default setting is to run the spikes implementation of BASiCS. The no-spikes implementation can be used by setting *WithSpikes = FALSE* in the call to *BASiCS_MCMC*. To run the regression BASiCS model, the user can set *Regression = TRUE* in the call to *BASiCS_MCMC* and *Regression = FALSE* to run the non-regression BASiCS model.

#### The Horizontal Integration Approach

As seen in Figure 4A, BASiCS (Vallejos et al., 2015, Vallejos et al., 2016) builds upon a vertical integration framework, exploiting a set of spike-in sequences (e.g. the set of 92 ERCC molecules described in Jiang et al., 2011) as a *gold standard* to aid normalisation and to quantify technical artifacts. However, while the addition of spike-in genes prior to sequencing is theoretically appealing (Lun et al., 2017), several practical limitations affect their utility (Vallejos et al., 2017). For example, the addition of spike-ins is not trivial in droplet-based protocols such as those introduced by Klein et al. (2015) and Macosko et al. (2015).

Here, we extend BASiCS to not rely on spike-in genes using principles of measurement error models where — in the absence of gold standard features — technical variation is quantified through *replication* (Carroll, 1998). As scRNA-seq is a destructive technology, it is not possible to replicate experiments by sequencing the same cells multiple times. However, we rely on the replication of population-level characteristics of the cells through appropriate experimental design (Tung et al., 2017) by randomly allocating cells from the same population to multiple independent experimental replicates (hereafter these are referred to as *batches*). Given such an experimental design, we assume that biological effects are shared across batches and that technical variation will be reflected by spurious differences between cells and batches.

#### The Horizontal Integration Model

Following this reasoning, we use a horizontal data integration approach to leverage information from multiple batches of sequenced cells to estimate biological effects that are not confounded by technical variation (see Figure 4B). Let *X*_*ijk*_ be a random variable representing the count (read- or UMI-based) for gene *i* (∈{1,…,q}) in cell *j* $\left(\in \left\{1,\ldots ,n_{k}\right\}\right)$ of the *k*-th batch (k∈{1,…,K}). The following model is proposed:

(Equation 37) $$X_{ijk}|\mu_{i},\nu_{jk},\rho_{ijk}\overset{\text{ind}}{∼}\text{Poisson}\left(\nu_{jk}\mu_{i}\rho_{ijk}\right),$$

(Equation 38) $$\text{with}\;\nu_{jk}|s_{jk},\theta \overset{\text{ind}}{∼}\text{Gamma}\left(1/\theta_{k},1/\left(s_{jk}\theta_{k}\right)\right)\;\text{and}\;\rho_{ijk}|\delta_{i}\overset{\text{ind}}{∼}\text{Gamma}\left(1/\delta_{i},1/\delta_{i}\right).$$

A key assumption underlying this model is that biological effects ($\mu_{i}$ and $\delta_{i}$) are shared across all batches and, therefore, we borrow information across cells in all batches to infer these parameters. In contrast to the original implementation of BASiCS, the absence of spike-in genes prevents the definition of two separate normalisation effects to capture nuisance differences in the scale of the observed read-counts between cells: one to capture differences in cellular mRNA content, one to capture technical artefacts (e.g. sequencing depth). Instead, in Equation 38, the normalisation parameters *s*_*jk*_ capture a combination of these effects. The latter are inferred by borrowing information across all genes assuming that $E\left(s_{jk}\right)=1$ *a priori*. Residual technical over-dispersion that is not captured by these normalisation parameters is captured by batch-specific parameters *θ*_*k*_.

Based on the proportion of variability that is attributed to a biological component, our model can be used to identify highly and lowly variable genes within a population of cells (see Vallejos et al., 2015). Moreover, differences in mean and over-dispersion between cell populations can be highlighted by comparing gene-specific parameters ($\mu_{i}$, $\delta_{i}$). Finally, when adopting the prior specification described for the regression BASiCS model, our model can also be used to compare transcriptional heterogeneity in terms of a residual over-dispersion parameters $\epsilon_{i}$.

#### Identifiability and Prior Specification

The model in Equation 37 and Equation 38 is not identifiable, i.e. the scale of cell-specific normalisation parameters *s*_*jk*_ and gene-specific mean expression parameters $\mu_{i}$ cannot be separately estimated from the data. As a solution, the following identifiability restriction is proposed:

(Equation 39) $${\left(\prod_{i=1}^{q}\mu_{i}\right)}^{1/q}=\mu_{0}\Leftrightarrow \frac{1}{q}\sum_{i=1}^{q}\log\left(\mu_{i}\right)=\;\log\left(\mu_{0}\right),\;\text{for}\;\text{a}\;\text{fixed}\;\text{known}\;\mu_{\text{0}}\text{.}$$

In Equation 39, the geometric mean of mean expression parameters $\mu_{i}$ is fixed (when analysing multiple populations, this restriction independently applies within each population). In practice, we replace the value of $\mu_{0}$ by its empirical counterpart, e.g. adopting the normalization strategy implemented in Lun et al. (2016). To avoid ill-defined situations, this calculation must exclude genes with zero total counts across all cells (for which the empirical estimate of $\mu_{i}$ is equal to 0). We note, however, that the actual value of $\mu_{0}$ is not critical, as global offset effects between cell populations can be corrected post hoc (see Vallejos et al., 2016).

Marginally, we assign a log-Normal $\left(0,s_{\mu }^{2}\right)$ prior distribution to each $\mu_{i}$. However, we do not assume these parameters to be *a priori* independent. Instead, an appropriate correlation structure is introduced to satisfy the identifiability restriction in Equation 39. Following Theorem 8.2 in West and Harrison (1989), this correlated prior is defined as

(Equation 40) $$\log\left(\mu \right)={\left(\log\left(\mu_{1}\right),\ldots ,\;\log\left(\mu_{q}\right)\right)}^{\prime }∼{\text{N}}_{q}\left(\log\left(\mu_{0}\right)1_{q},a_{\mu }^{2}\left(I_{q}-1_{q}1_{q}^{\prime }/q\right)\right),$$

where *q* is the number of genes, **1***_q_* denotes a *q*-dimensional vector of ones and **I**_*q*_ denotes a *q*-dimensional identity matrix. Due to the identifiability constraint in Equation 39, the covariance matrix in Equation 40 is not full rank. Hence, for an arbitrarily chosen *reference* gene *r*, Equation 40 can be factorised as a multivariate normal prior for $\log\left(\mu_{-r}\right)={\left(\log\left(\mu_{1}\right),\ldots ,\;\log\left(\mu_{r-1}\right),\;\log\left(\mu_{r+1}\right),\ldots ,\;\log\left(\mu_{q}\right)\right)}^{'}$ and a point mass prior for $\log\left(\mu_{r}\right)|\log\left(\mu_{-r}\right)$ (see Proposition 2). As a result, posterior inference can be implemented by drawing posterior samples for $\log\left(\mu_{-r}\right)$, leaving posterior samples for $\log\left(\mu_{r}\right)$ to be completely specified by the identifiability restriction.

#### Using a Stochastic Reference Gene

The vertical integration version of BASiCS (with spike-ins) is used as a benchmark for the model in Equation 37 and Equation 38. To illustrate its performance, we use the dataset of Grün et al. (2014), for which technical spike-ins and multiple batches of sequenced cells are available. In both cases, the MCMC sampler was run for 20,000 iterations, storing draws every 10 iterations and ignoring an initial burn-in period of 10,000 iterations (hence, results are shown in terms of 1,000 iterations).

Overall, posterior inference is unaffected for the majority of genes (Figures 4C and 4D). However, as it can be expected, the effect of the prior is more prominent for lowly expressed genes where the data is less informative. In those cases, the identifiability constrain in Equation 39 slightly shrinks posterior estimates of mean expression parameters $\mu_{i}$ towards $\mu_{0}$. We observe that posterior inference is distorted for the arbitrarily chosen reference gene (see Figures S4A and S4B). To overcome this problem, we introduce the use of a stochastic reference choice. The latter randomly selects a reference gene at each iteration of the MCMC algorithm. As a result, each gene is treated as reference only a small proportion of times, leading to valid posterior inference for all genes (see Figures S4C and S4D).

#### Technical Details

##### A Correlated Prior to Satisfy the Identifiability Restriction

*Proposition 1.* The prior distribution

(Equation 41) $$\mu_{i}∼\text{log}\text{-}\text{N}\left(0,a_{\mu }^{2}\right),\;subject\;to\;{\left(\prod_{i=1}^{q}\mu_{i}\right)}^{1/q}=\mu_{0}\left(for\;fixed\;\mu_{0}\right)$$

is equivalent to

(Equation 42) $$\log\left(\mu \right)={\left(\log\left(\mu_{1}\right),\ldots ,\;\log\left(\mu_{q}\right)\right)}^{\prime }∼{\text{N}}_{q}\left(\log\left(\mu_{0}\right)1_{q},a_{\mu }^{2}\left(I_{q}-1_{q}1_{q}^{\prime }/q\right)\right),$$

where **1**_q_ denotes a q-dimensional vector of ones and **I**_q_ denotes a q-dimensional identity matrix.

*Proof.* The proof follows the same steps as in the proof of Theorem 8.2 in West and Harrison (1989). Let $M=\sum_{i=1}^{q}\log\left(\mu_{i}\right)$. It can be shown that

(Equation 43) $$\begin{array}{c} \left(\begin{array}{c} \log\;\left(\mu \right)\\ M \end{array}\right)∼{\text{N}}_{q+1}\left(0_{q+1},\left(\begin{array}{cc} a_{\mu }^{2}I_{q} & a_{\mu }^{2}1_{q}\\ a_{\mu }^{2}{1^{\prime }}_{q} & a_{\mu }^{2}{1^{\prime }}_{q}1_{q} \end{array}\right)\right)\\ \equiv {\text{N}}_{q+1}\left(0_{q+1},\left(\begin{array}{cc} a_{\mu }^{2}{\text{I}}_{q} & a_{\mu }^{2}1_{q}\\ a_{\mu }^{2}{1^{\prime }}_{q} & a_{\mu }^{2}q \end{array}\right)\right). \end{array}$$

Hence

(Equation 44) $$\log\left(\mu \right)|M∼{\text{N}}_{q}\left(\left(M/q\right)1_{q},a_{\mu }^{2}\left({\text{I}}_{q}-1_{q}1_{q}^{\prime }/q\right)\right).$$

Finally, replacing $M=q\;\log\left(\mu_{0}\right)$, we obtain

(Equation 45) $$\log\left(\mu \right)|\left(M=q\;\log\left(\mu_{0}\right)\right)∼{\text{N}}_{q}\left(\log\left(\mu_{0}\right)1_{q},a_{\mu }^{2}\left({\text{I}}_{q}-1_{q}1_{q}^{\prime }/q\right)\right).$$

*Proposition 2*. Let $\log\left(\mu_{-r}\right)\equiv {\left(\log\left(\mu_{1}\right),\ldots ,\;\log\left(\mu_{r-1}\right),\;\log\left(\mu_{r+1}\right),\ldots ,\;\log\left(\mu_{q}\right)\right)}^{\prime }$, where r (1≤r≤q) denotes an arbitrarily chosen reference gene. The correlated prior derived in Proposition 1 can be factorized in terms of a multivariate normal prior for $\log\left(\mu_{-r}\right)$ and a point mass prior for $\log\left(\mu_{r}\right)|\log\left(\mu_{-r}\right)$ which is located at $q\;\log\left(\mu_{0}\right)-\sum_{i\neq r}\log\left(\mu_{i}\right)$.

*Proof.* Standard multivariate normal theory leads to

(Equation 46) $$\log\left(\mu_{-r}\right)∼{\text{N}}_{q-1}\left(\log\left(\mu_{0}\right)1_{q-1},a_{\mu }^{2}\left({\text{I}}_{q-1}-1_{q-1}1_{q-1}^{\prime }/q\right)\right),$$

and

(Equation 47) $$\log\left(\mu_{r}\right)|\log\left(\mu_{-r}\right)∼{\text{N}}_{1}\left(m,\Sigma \right),$$

with

(Equation 48) $$\begin{array}{c} m=\;\log\left(\mu_{0}\right)+\left(-1_{q-1}^{\prime }/q\right){\left({\text{I}}_{q-1}-1_{q-1}1_{q-1}^{\prime }/q\right)}^{-1}\left(\log\left(\mu_{-r}\right)-\log\left(\mu_{0}\right)1_{q-1}\right)\\ =\;\log\left(\mu_{0}\right)-1_{q-1}^{\prime }\left({\text{I}}_{q-1}+1_{q-1}1_{q-1}^{\prime }\right)\left(\log\left(\mu_{-r}\right)-\log\left(\mu_{0}\right)1_{q-1}\right)/q\\ \;\;\;\;\;\;\left(\text{see}\;\text{Miller,}\;1981\right)\\ =\;\log\left(\mu_{0}\right)-q1_{q-1}^{\prime }\left(\log\left(\mu_{-r}\right)-\log\left(\mu_{0}\right)1_{q-1}\right)/q\\ =\log\left(\mu_{0}\right)-\sum_{i\neq r}\log\left(\mu_{i}\right)+\left(q-1\right)\log\left(\mu_{0}\right)\\ =q\;\log\left(\mu_{0}\right)-\sum_{i\neq r}\log\left(\mu_{i}\right) \end{array},$$

and

(Equation 49) $$\begin{array}{c} \Sigma \propto \left(1-1/q\right)-\left(-1_{q-1}^{\prime }/q\right){\left({\text{I}}_{q-1}-1_{q-1}1_{q-1}^{\prime }/q\right)}^{-1}\left(1_{q-1}/q\right)\\ =\left(1-\frac{1}{q}\right)-\frac{1}{q^{2}}1_{q-1}^{\prime }\left({\text{I}}_{q-1}+1_{q-1}{1^{\prime }}_{q-1}\right)1_{q-1}\left(\text{see}\;\text{Miller,}\;1981\right)\\ =\left(1-\frac{1}{q}\right)-\frac{1}{q^{2}}1_{q-1}^{\prime }\left(1_{q-1}+\left(q-1\right)1_{q-1}\right)\\ =\left(1-\frac{1}{q}\right)-\frac{1}{q^{2}}q1_{q-1}^{\prime }1_{q-1}\equiv 0 \end{array}.$$

*Proposition 3*. Under the same assumptions as in Proposition 1. Let $\mu_{-i,r}$ be the vector obtained after removing elements i and r from ${\left(\mu_{1},\ldots ,\mu_{q}\right)}^{\prime }$. It can be shown that

(Equation 50) $$\log\left(\mu_{i}\right)|\log\left(\mu_{-i,r}\right)∼N\left(\frac{1}{2}\left(q\;\log\left(\mu_{0}\right)-1_{q-2}^{\prime }\;\log\left(\mu_{-i,r}\right)\right),\frac{1}{2}a_{\mu }^{2}\right),$$

where **1**_q−2_ denotes a (q−2) -dimensional vector of ones.

*Proof.* Standard multivariate normal theory leads to

(Equation 51) $$\log\left(\mu_{i}\right)|\log\left(\mu_{-i,r}\right)∼{\text{N}}_{1}\left(m,\Sigma \right),$$

with

(Equation 52) $$\begin{array}{c} m=\;\log\left(\mu_{0}\right)+\left(-1_{q-2}^{\prime }/q\right){\left({\text{I}}_{q-2}-1_{q-2}1_{q-2}^{\prime }/q\right)}^{-1}\left(\log\left(\mu_{-i,r}\right)-\log\left(\mu_{0}\right)1_{q-2}\right)\\ =\;\log\left(\mu_{0}\right)-1_{q-2}^{\prime }\left({\text{I}}_{q-2}+\frac{1}{2}1_{q-2}1_{q-2}^{\prime }\right)\left(\log\left(\mu_{-i,r}\right)-\log\left(\mu_{0}\right)1_{q-2}\right)/q\\ \;\;\;\;\;\left(\text{see}\;\text{Miller,}\;1981\right)\\ =\;\log\left(\mu_{0}\right)-\frac{1}{2}\left(1_{q-2}^{\prime }\;\log\left(\mu_{-i,r}\right)-\left(q-2\right)\log\left(\mu_{0}\right)\right)\\ =\frac{q}{2}\log\left(\mu_{0}\right)-\frac{1}{2}1_{q-2}^{\prime }\;\log\left(\mu_{-i,r}\right) \end{array},$$

and

(Equation 53) $$\begin{array}{c} \Sigma =a_{\mu }^{2}\left(\left(1-1/q\right)-\left(-1_{q-2}^{\prime }/q\right){\left({\text{I}}_{q-2}-1_{q-2}1_{q-2}^{\prime }/q\right)}^{-1}\left(1_{q-2}/q\right)\right)\\ =a_{\mu }^{2}\left(\left(1-\frac{1}{q}\right)-\frac{1}{q^{2}}1_{q-2}^{\prime }\left({\text{I}}_{q-2}+\frac{1}{2}1_{q-2}1_{q-2}^{\prime }\right)1_{q-2}\right)\left(\text{see}\;\text{Miller,}\;1981\right)\\ =a_{\mu }^{2}\left(\left(1-\frac{1}{q}\right)-\frac{1}{q^{2}}1_{q-2}^{\prime }\left(1_{q-2}+\frac{q-2}{2}1_{q-2}\right)\right)\\ =\frac{1}{2}a_{\mu }^{2} \end{array}$$

#### Implementation

Bayesian inference is implemented using an adaptive Metropolis within Gibbs algorithm (Roberts and Rosenthal, 2009). After integrating out the random effects $\rho_{ijk}$, the full conditionals required for this implementation are based on the following likelihood function:

(Equation 54) $$\begin{array}{l} \left[\prod_{i=1}^{q}\prod_{k=1}^{K}\prod_{j=1}^{n_{k}}\frac{\Gamma \left(x_{ijk}+\frac{1}{\delta_{i}}\right)}{\Gamma \left(\frac{1}{\delta_{i}}\right)x_{ijk}!}{\left(\frac{\frac{1}{\delta_{i}}}{\nu_{jk}\mu_{i}+\frac{1}{\delta_{i}}}\right)}^{\frac{1}{\delta_{i}}}{\left(\frac{\nu_{jk}\mu_{i}}{\nu_{jk}\mu_{i}+\frac{1}{\delta_{i}}}\right)}^{x_{ijk}}\right]\\ \times \left[\prod_{k=1}^{K}\prod_{j=1}^{n_{k}}\frac{{\left(s_{jk}\theta_{k}\right)}^{-\frac{1}{\theta_{k}}}}{\Gamma \left(\frac{1}{\theta_{k}}\right)}\nu_{jk}^{\frac{1}{\theta_{k}}-1}\;\exp\left\{-\frac{\nu_{jk}}{s_{jk}\theta_{k}}\right\}\right]. \end{array}$$

Let *r* denote an arbitrarily chosen reference gene. If $\mu_{i}$ and $\delta_{i}$ are assumed to be *a priori* independent (i.e. as in Vallejos et al., 2016), the associated full conditionals for $\mu_{i}$ (*i*≠*r*) are given by:

(Equation 55) $$\pi \left(\mu_{i}|\mu_{-i,r},\cdot \right)\propto \frac{\mu_{i}^{\sum_{k=1}^{K}\sum_{j=1}^{n_{k}}x_{ijk}}}{\prod_{k=1}^{K}\prod_{j=1}^{n_{k}}{\left(\nu_{jk}\mu_{i}+1/\delta_{i}\right)}^{x_{ijk}+1/\delta_{i}}}\times \pi \left(\mu_{i}|\mu_{-i,r}\right),$$

where $\pi \left(\mu_{i}|\mu_{-i,r}\right)$ is defined as in Proposition 3 and $\mu_{-i,r}$ is the vector obtained after removing elements *i* and *r* from ${\left(\mu_{1},\ldots ,\mu_{q}\right)}^{\prime }$. Due to the identifiability constraint, $\mu_{r}|\mu_{-r}\equiv \mu_{0}^{q}{\left(\prod_{i\neq r}\mu_{i}\right)}^{-1}$ with probability 1. If a gene *i* (*i*≠*r*) is excluded from the identifiability constraint (genes with less than 1 count per cell [on average] are excluded), Equation 55 becomes

(Equation 56) $$\pi \left(\mu_{i}|\mu_{-i,r},\cdot \right)\propto \frac{\mu_{i}^{\sum_{k=1}^{K}\sum_{j=1}^{n_{k}}x_{ijk}}}{\prod_{k=1}^{K}\prod_{j=1}^{n_{k}}{\left(\nu_{jk}\mu_{i}+1/\delta_{i}\right)}^{x_{ijk}+1/\delta_{i}}}\times \exp\left\{-\frac{1}{2a_{\mu }^{2}}{\left(\log\left(\mu_{i}\right)\right)}^{2}\right\}\frac{1}{\mu_{i}}.$$

Under this prior, the remaining full conditionals are given by:

(Equation 57) $$\pi \left(\delta_{i}|\cdot \right)\propto \frac{\delta_{i}^{-\left(n/\delta_{i}\right)}}{\Gamma^{n}\left(1/\delta_{i}\right)}\left[\prod_{k=1}^{K}\prod_{j=1}^{n_{k}}\frac{\Gamma \left(x_{ijk}+1/\delta_{i}\right)}{{\left(\nu_{jk}\mu_{i}+1/\delta_{i}\right)}^{x_{ijk}+1/\delta_{i}}}\right]\exp\left\{-\frac{1}{2a_{\delta }^{2}}{\left(\log\left(\delta_{i}\right)\right)}^{2}\right\}\frac{1}{\delta_{i}},$$

(Equation 58) $$\pi \left(s_{jk}|\cdots \right)\propto {\left(s_{jk}\right)}^{a_{s}-\left(1/\theta_{k}\right)-1}\;\exp\left\{-\frac{\nu_{jk}}{s_{jk}\theta_{k}}-s_{jk}b_{s}\right\},$$

(Equation 59) $$\pi \left(\nu_{jk}|\cdot \right)\propto \frac{\nu_{jk}^{\sum_{i=1}^{q}x_{ijk}+1/\theta_{k}-1}}{\prod_{i=1}^{q}{\left(\nu_{jk}\mu_{i}+1/\delta_{i}\right)}^{x_{ijk}+1/\delta_{i}}}e^{-\nu_{jk}/\left(\theta_{k}s_{jk}\right)},$$

(Equation 60) $$\pi \left(\theta_{k}|\cdot \right)\propto \frac{{\left(\prod_{j=1}^{n_{k}}\left(\nu_{jk}/s_{jk}\right)\right)}^{1/\theta_{k}}}{\Gamma^{n_{k}}\left(1/\theta_{k}\right)}\theta_{k}^{a_{\theta }-\left(n_{k}/\theta_{k}\right)-1}e^{-\left(1/\theta_{k}\right)\sum_{j=1}^{n_{k}}\left(\nu_{jk}/s_{jk}\right)-b_{\theta }\theta_{k}},$$

where $n=\sum_{k=1}^{K}n_{k}$. Alternatively, if the joint informative prior is adopted, Equation 55 and Equation 57 are respectively replaced by

(Equation 61) $$\begin{array}{c} \pi \left(\mu_{i}|\mu_{-i,r},\cdot \right)\propto \frac{\mu_{i}^{\sum_{k=1}^{K}\sum_{j=1}^{n_{k}}x_{ijk}}}{\prod_{k=1}^{K}\prod_{j=1}^{n_{k}}{\left(\nu_{jk}\mu_{i}+1/\delta_{i}\right)}^{x_{ijk}+1/\delta_{i}}}\times \pi \left(\mu_{i}|\mu_{-i,r}\right)\times \\ \exp\left\{-\frac{{\left(\log\left(\mu_{i}\right)\right)}^{2}}{2a_{\mu }^{2}}-\frac{{\left(\log\left(\delta_{i}\right)-f\left(\mu_{i}\right)\right)}^{2}}{2\sigma^{2}/\lambda_{i}}\right\}\frac{1}{\mu_{i}} \end{array},$$

(Equation 62) $$\begin{array}{c} \pi \left(\delta_{i}|\cdot \right)\propto \frac{\delta_{i}^{-\left(n/\delta_{i}\right)}}{\Gamma^{n}\left(1/\delta_{i}\right)}\left[\prod_{k=1}^{K}\prod_{j=1}^{n_{k}}\frac{\Gamma \left(x_{ijk}+1/\delta_{i}\right)}{{\left(\nu_{jk}\mu_{i}+1/\delta_{i}\right)}^{x_{ijk}+1/\delta_{i}}}\right]\times \\ \exp\left\{-\frac{{\left(\log\left(\delta_{i}\right)-f\left(\mu_{i}\right)\right)}^{2}}{2\sigma^{2}/\lambda_{i}}\right\}\frac{1}{\delta_{i}}, \end{array}.$$

### Quantification and Statistical Analysis

#### Quality Filtering of Single Cell RNA Sequencing Data

We employed a range of different datasets to test the proposed methodology. These datasets were selected to cover different experimental techniques (with and without unique molecular identifiers, UMI) and to encompass a variety of cell populations. Moreover, key features of each dataset can be found in Table S1.

#### *Dictyostelium* Cells

Antolović et al. (2017) studied changes in expression variability between 0 hours (undifferentiated), 3 hours and 6 hours of *Dictyostelium* differentiation. Raw data is available by direct download (see Data S1 in Antolović et al., 2017). Across all time-points, 5 cells were removed due to low quality. Technical spike-in genes that were not detected and biological genes with an average expression (across all cells) smaller than 1 count were removed. In total, 433 cells (131 cells and 3 batches at 0h, 157 cells and 3 batches at 3h, and 145 cells and 3 batches at 6h) and 10551 genes (88 technical and 10650 biological genes) passed filtering. We used data from the 0h time point to test the functionality of our model.

#### Mouse Brain Cells

This dataset was composed of UMI scRNA-seq data of cells isolated from the mouse somatosensory cortex and hippocampal CA1 region (Zeisel et al., 2015). Raw data is available from Gene Expression Omnibus under accession code GEO: GSE60361. Prior to the analysis, we removed technical genes with 0 total counts and biological genes for which the average count across all 3007 cells was below 0.1. The groups comprising microglia cells and CA1 neurons were chosen to be analysed. For these groups, 98 cells (microglia), 939 cells (CA1 pyramidal neurons) and 10744 genes (10687 biological and 57 technical genes) were left to be analysed.

#### Pool-and-Split RNA-Seq Data

This UMI-based dataset provides a control experiment to assess changes in biological heterogeneity in a situation where mean expression remains unchanged across conditions. Pool-and-split samples were created by pooling 1 million mESCs grown in 2i or serum medium and splitting 20pg of RNA into aliquots. These libraries are compared against single-cell samples (mESCs) (Grün et al., 2014). Raw data is available from Gene Expression Omnibus under accession code GEO: GSE54695.

As in Grün et al. (2014), some cells were removed from the analysis due to low expression of the stem cell marker Oct4. Technical genes with 0 total counts were also removed from the analysis. Additionally, lowly expressed biological genes with fewer than 0.5 counts (on average, across all samples) were excluded. This left 258 libraries (74 single mESCs grown in 2i medium, 52 single mESCs grown in serum medium, 76 pool-and-split aliquots from cells grown in 2i medium and 56 pool-and-split aliquots from cells grown in serum medium) as well as 8924 genes (50 technical spike-ins and 8874 biological genes) for the analysis. Each condition contained 2 batches.

Matched single molecule fluorescence in situ hybridization (smFISH) data from mESCs grown in 2i and serum media were obtained from Dominic Grün (Max Planck Institute of Immunobiology and Epigenetics, Freiburg, Germany) through personal communications. This smFISH experiment assayed 9 genes (*Gli1*, *Klf4*, *Notch1*, *Pcna*, *Pou5f1*, *Sohlh2*, *Sox2*, *Stag3*, *Tpx2*) in more than 70 cells per condition. We excluded *Notch1* from the analysis due to strong disagreement between smFISH and scRNA-seq data of cells grown in serum medium.

#### CD4^+^ T Cells

Non-UMI scRNA-seq data of CD4^+^ T cells were taken from Martinez-Jimenez et al. (2017). Raw data are available from ArrayExpress under accession code ArrayExpress: E-MTAB-4888. To perform a variety of tests, naive and activated CD4^+^ T cells from young *Mus musculus* (B6) mice were selected. Biological genes with an average count < 1 and non-detected technical genes were removed from the analysis. In total, 146 cells (93 naive and 53 activated CD4^+^ T cells) and 10553 genes (10495 biological and 58 technical genes) passed filtering. Each condition contains 2 replicates.

#### CD4^+^ T Cell Differentiation

Non-UMI scRNA-seq data were generated from CD4^+^ T cells during differentiation towards Th1 and Tfh cell fates after *Plasmodium* infection (Lönnberg et al., 2017). Raw reads were downloaded from ArrayExpress [ArrayExpress: E-MTAB-4388] and mapped against the *Mus musculus* genome (mm10) using *gsnap* (Wu and Nacu, 2010) with default settings. Read counting was performed using *HTSeq* (Anders et al., 2015) with default settings.

Quality control was performed by removing cells with fewer than 300,000 biological reads or fewer than 600,000 technical reads at day 2. At day 4 and 7, cells with fewer than 1,000,000 biological reads were excluded from downstream analysis. Additionally, we removed genes that did not show an average detection of more than 1 read at day 2, day 3, day 4 or day 7 after infection. After applying these criteria, 376 cells (Day 0: 16 cells, Day 2: 89, Day 3: 21, Day 4: 133, Day 7: 64, Day 7 non-infected: 53) and 7899 genes (7847 biological and 52 technical) remained for analysis. Note that, due to low sample sizes, we focused our analysis on data from day 2, day 4 and day 7 post-infection.

#### Thresholds When Assessing Expression Changes

Statistical assessment of changes in mean expression and residual over-dispersion was performed between datasets using the regression BASiCS model. Unless otherwise indicated, the tolerance threshold was set to $\tau_{0}=\;\log_{2}\left(1.5\right)=0.58$ for differential mean expression testing, $\omega_{0}=\;\log_{2}\left(1.5\right)=0.58$ for differential over-dispersion testing and to $\psi_{0}=0.41$ for differential residual over-dispersion testing. The expected false discovery rate was controlled to 10%. This information is also displayed in figure legends.

#### Functional Annotation Analysis

We performed functional annotation analysis using DAVID version 6.8 (Dennis et al., 2003). All genes considered for differential testing were used as background. The functional annotation clustering function in DAVID was used to cluster annotation categories based on similarity and to sort them according to their enrichment score.

#### Stabilization of Posterior Inference for Small Sample Sizes

To compare parameter estimates of the regression and non-regression model across different sample sizes, we used the CA1 pyramidal neuron population from Zeisel et al. (2015). The regression BASiCS model was first run on the full population of 939 cells to generate *pseudo* ground truth parameter estimates. Subsequently, 50, 100, 150, 200, 250, 300 and 500 cells were randomly sub-sampled from the full population prior to parameter estimation. This procedure was repeated 10 times for each sample size. Based on parameter estimates using the non-regression model, we split the genes into three sets: lowly expressed $\left(\mu_{i}<1.89\right)$, medium expressed $\left(1.89<\mu_{i}<5.37\right)$ and highly expressed $\left(\mu_{i}>5.37\right)$. These cut-off values were chosen such that a third of genes classifies into each category. We dissected the results of this experiment in three ways. First, we visualize boxplots showing all estimates of gene-specific parameters for a single sub-sampling experiment (Figure 3). Second, we computed the log_2_ fold change for estimates of gene-specific over-dispersion parameters δ_*i*_ between the regression and non-regression BASiCS models (Figures S3A–S3C). Third, for each sub-sampling experiment, sample size and gene set, we computed the median log_2_ fold change in μ_*i*_ and δ_*i*_ and the median difference for $\epsilon_{i}$ between estimates and the *pseudo* ground truth. The median and the range of these values across 10 sub-sampling experiment is used for visualization purposes (see Figure S3D–S3F).

External validation for posterior estimates of gene-specific model parameters was obtained using matched scRNA-seq and smFISH data of mouse embryonic stem cells grown in 2i and serum media (see Table S1 and Grün et al., 2014). As in Brennecke et al. (2013), to calculate residual CV^2^ values for the smFISH data, we defined residuals obtained after fitting a gamma generalized linear model with an identity link (*glmgam.fit* of the *statmod* package in R) between the CV^2^ and the reciprocal log-transformed mean transcript counts.

#### Changes in Variability during CD4^+^ T Cell Activation

Firstly, we compare the results obtained by the regression BASiCS model with respect those presented in Martinez-Jimenez et al. (2017). To allow a direct comparison of the results, the same inclusion criteria as in Martinez-Jimenez et al. (2017) is adopted, i.e. we excluded genes with low mean expression $\left(\mu_{i}<50\right)$ in both conditions from testing. Moreover, our minimum tolerance thresholds were also adapted to match the choices in Martinez-Jimenez et al. (2017). To detect differentially expressed genes (mean) a minimum tolerance threshold $\tau_{0}=2$ was used (see Figure S5A). To compare the detection of differentially over-dispersed genes, we performed differential mean expression testing using a stringent minimum tolerance threshold $\tau_{0}=0$ for both models (this is to avoid the results being confounded by changes in mean, see upper panel in Figure S5B). For the 463 genes that are detected as non-differentially expressed by both models for this threshold, a total of 111 genes are detected as differentially over-dispersed by either model (minimum tolerance log_2_ fold change threshold $\omega_{0}=\;\log_{2}\left(1.5\right)=0.58$). Out of this set, 93 genes (∼83%) are detected as differentially over-dispersed by both models (see lower panel in Figure S5B)).

In this article, we exclude genes whose estimated mean expression parameters μ_*i*_ was below 1 from the differential testing. Furthermore, a log_2_ fold change threshold $\tau_{0}=1$ was adopted for mean expression testing. Unlike the more stringent threshold used by Martinez-Jimenez et al. (2017) $\left(\tau_{0}=2\right)$, this choice allows us to detect more subtle changes in mean expression. Moreover, the default threshold $\psi_{0}=0.41$ was used for differential variability testing. The expected false discovery rate (EFDR) was controlled to 10%.

Genes were sorted into four categories based on their changes in variability and mean expression: down-regulated upon activation with (i) lower and (ii) higher variability, and up-regulated with (iii) lower and (iv) higher variability (see Figure 5A). For each of these gene sets, functional annotation analysis was performed using all tested genes as background. The functional annotation clustering tool in DAVID (Dennis et al., 2003) was used to cluster annotation categories based on similarity and to sort them according to their enrichment score. Here, we list the top 3 functional annotation clusters per gene set and their corresponding enrichment score (ES):

• Down-regulated with lower variability: Pleckstrin homology domain (ES = 1.57), G protein signalling (ES = 1.51), glycosidase (ES = 1.49),
• Down-regulated with higher variability: Ankyrin repeat-containing domain (ES = 2.19), GTPase mediated signalling (ES = 1.51), steroid biosynthesis (ES = 0.89),
• Up-regulated with lower variability: RNA polymerase (ES = 1.6), RNA binding (ES = 1.53), splicing (ES = 1.41),
• Up-regulated with higher variability: Cytokine-cytokine receptor interaction (ES = 1.65), WD40 repeat (ES = 1.22), transcription (ES = 1.18).

To visualize gene expression in individual cells, we denoised the raw expression counts using the *BASiCS_DenoisedCounts* function.

Finally, we performed a synthetic experiment to illustrate how individual cells that highly express certain genes can drive the detection of changes in variability. For this purpose, we created a mixed population of cells by combining 5 activated CD4^+^ T cells with a population of 93 naive CD4^+^ T cells. In this mixture, response genes are lowly expressed on average and show expression outliers in a small subset of cells. *Il2* represents a gene with statistically significant higher mean expression and higher residual over-dispersion in the mixed population (see Figure S5C). All genes that show increased mean expression as well as increased residual over-dispersion are visualized in Figure S5D.

#### Changes in Variability during CD4^+^ T Cell Differentiation

To detect changes in over-dispersion and residual over-dispersion (variability) during CD4^+^ T cell differentiation, we performed two sets of tests between day 2 and day 4, day 4 and day 7, and day 2 and day 7. The minimum tolerance log_2_ fold change threshold to test changes in mean expression in the first test was set to $\tau_{0}=0$, while the threshold for the second test was set to $\tau_{0}=1$. The default threshold $\psi_{0}=0.41$ was used for differential variability testing. EFDR was controlled to 10%. To visualize gene expression in individual cells, we denoised the raw expression counts using the *BASiCS_DenoisedCounts* function.

The results of the first stringent test allow us to detect genes that do not change in mean expression between any of the three time points (126 genes). For these genes, the δ_*i*_ estimates are therefore comparable across the time points, avoiding the confounding with mean expression (see Figure 6A). To detect genes that show different variability patterns across the time points, we first removed all genes that are expressed in fewer than 2 cells in at least one time point. For the remaining genes, the second testing strategy was used and all genes with statistically significant changes in variability between day 2 and day 4, and day 4 and day 7 were collected (see Figure 6B). For analysis in Figures 6C and 6D the second testing strategy was used to detect changes in variability between day 2 and day 4.

Finally, we selected gene sets listed in Lönnberg et al. (2017) to visualize their changes in mean expression and residual over-dispersion. The first set of genes is taken from Figure 3E of the original publication, which filtered genes based on their association with the bifurcation of Th1 and Tfh differentiation. The second set of genes with sequential peak expression over pseudotime is taken from Figure 5A of the original publication, which were selected based on immunological relevance from a list of dynamic genes during *in vivo* differentiation (see Figure S6).

### Data and Software Availability

BASiCS is freely available as part of Bioconductor 3.7 (bioconductor.org).

The results displayed in this manuscript and its supplemental material use BASiCS version 1.1.57. All R scripts for data preparation and analysis are available at github.com/MarioniLab/RegressionBASiCS2017. This link also includes instructions to download all the publicly available datasets used throughout our analyses.

## Supplemental Information

Document S1. Figures S1–S6 and Table S1

Table S2. Differential Testing Results between Naive and Activated CD4+ T cells, Related to Figure 5 For each gene highlighted to have a statistically significant change in variability: estimated difference in residual over-dispersion parameters between active and naive CD4+ T cells (DistEpsilon), estimated log_2_ fold change in mean expression parameters between active and naive CD4+ T cells (Log2FCmu), and the result of the differential test (Regulation).

Document S2. Article plus Supplemental Information
