# Supplementary material for: Correcting the Mean-Variance Dependency for Differential Variability Testing Using Single-Cell RNA Sequencing Data
Source: Cell Syst. 2018 Sep 26;7(3):284–294.e12. doi: 10.1016/j.cels.2018.06.011 (PMC6167088; doi:10.1016/j.cels.2018.06.011)
Supplement: Document S1. Figures S1–S6 and Table S1 [file mmc1.pdf]

Cell Systems, Volume 7

Supplemental Information

Correcting the Mean-Variance Dependency

for Differential Variability Testing

Using Single-Cell RNA Sequencing Data

Nils Eling, Arianne C. Richard, Sylvia Richardson, John C. Marioni, and Catalina A. Vallejos

Supplemental figures

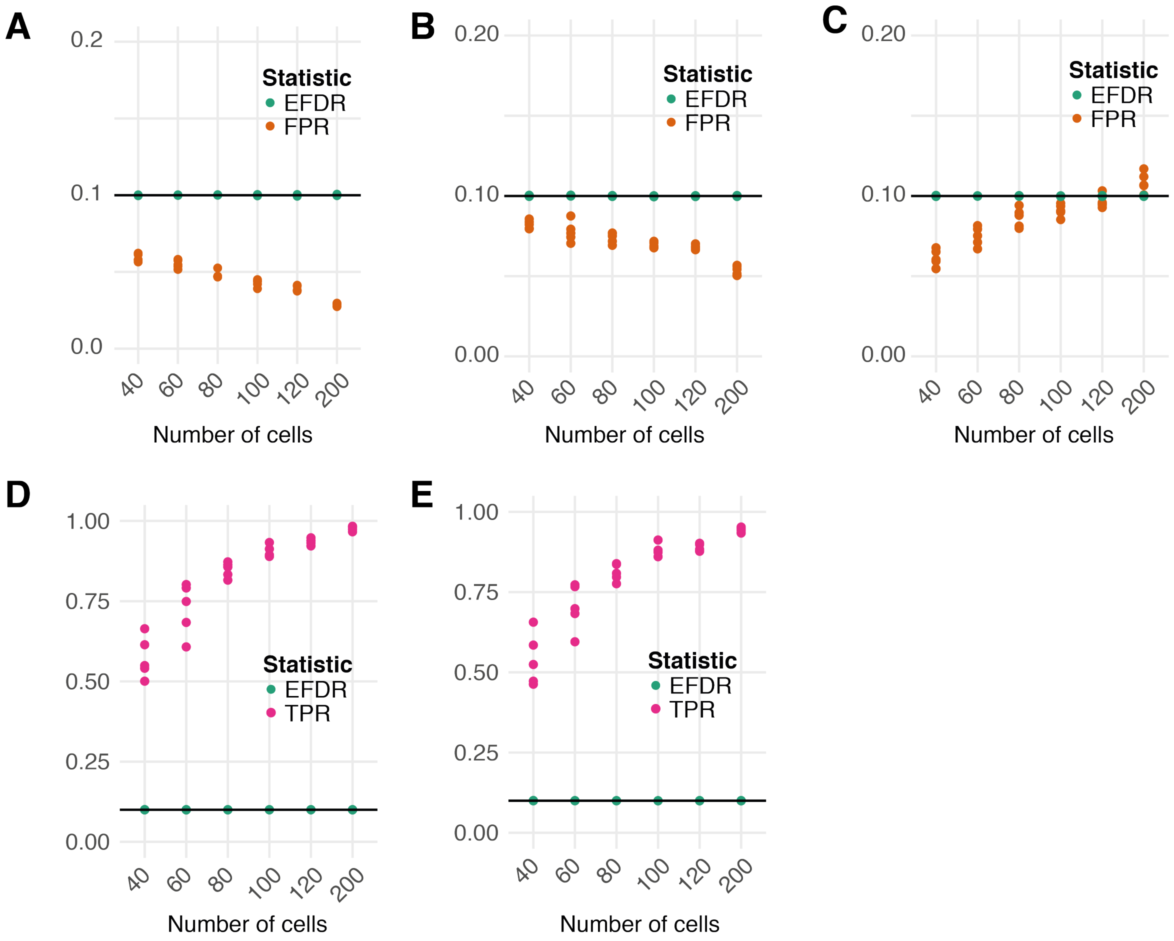

Figure S1: EFDR, FPR and TPR estimation using simulated data (Related to Figure 1). Data was simulated using the BASiCS model with model parameters set by empirical estimates based on on 98 microglia cells (see **STAR Methods**).

Different samples sizes (40 - 200 cells) were simulated in replicates of 5. Differential testing was performed between 2 simulated datasets of equal size to calculate the false positive rate (FPR, number of detections divided by number of genes tested), the true positive rate (number of true positive divided by number of positives). Moreover, we report the expected false discovery rate (EFDR, Newton et al., 2004). For each test, the EFDR was controlled to 10% and the default minimum tolerance thresholds were used ($\tau_0 = \log_2(1.5)$, $\omega_0 = \log_2(1.5)$ and $\psi_0 = 0.41$).

(A)-(C) Synthetic datasets generated using the null model (without changes in variability). FPR and EFDR for (A) differential mean expression, (B) differential over-dispersion and (C) differential residual over-dispersion testing using datasets with increasing samples sizes.

(D)-(E) Synthetic datasets generated using the alternative model where 1000 genes were randomly selected and their associated over-dispersion parameters were increased or decreased by a $\log_2$ fold change of 5 (see **STAR Methods**). TPR and EFDR for (D) differential over-dispersion testing and (E) differential residual over-dispersion testing using datasets with increasing samples sizes simulated.

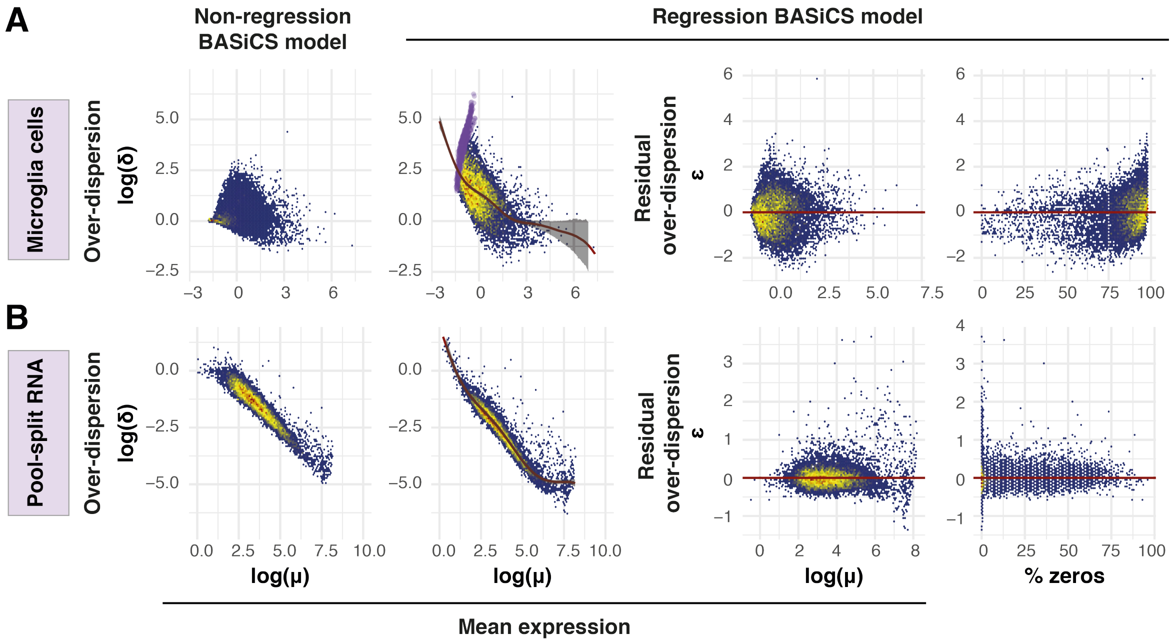

Figure S2: Parameter estimation using a scRNAseq and a technical pool-and-split RNA dataset (Related to Figure 2).

Model parameters were estimated using the regression and non-regression BASiCS models on (A) microglia cells (Zeisel et al., 2015) and (B) pool-and-split RNA (Grün et al., 2014). More details about these datasets are provided in **STAR Methods**. The colour code within the scatterplots is used to represent areas with high (yellow/red) and low (blue) concentration of genes.

First column: gene-specific over-dispersion δ_i versus mean expression μ_i as estimated by the non-regression BASiCS model.

Second column: gene-specific over-dispersion δ_i versus mean expression μ_i as estimated by the regression BASiCS model. The red line indicates the estimated regression trend. Purple dots indicate genes detected in less than 2 cells.

Third column: gene-specific residual over-dispersion ϵ_i versus mean expression μ_i as estimated by the regression BASiCS model.

Forth column: gene-specific posterior estimates for residual over-dispersion ϵ_i parameters versus percentage of zero counts for each gene.

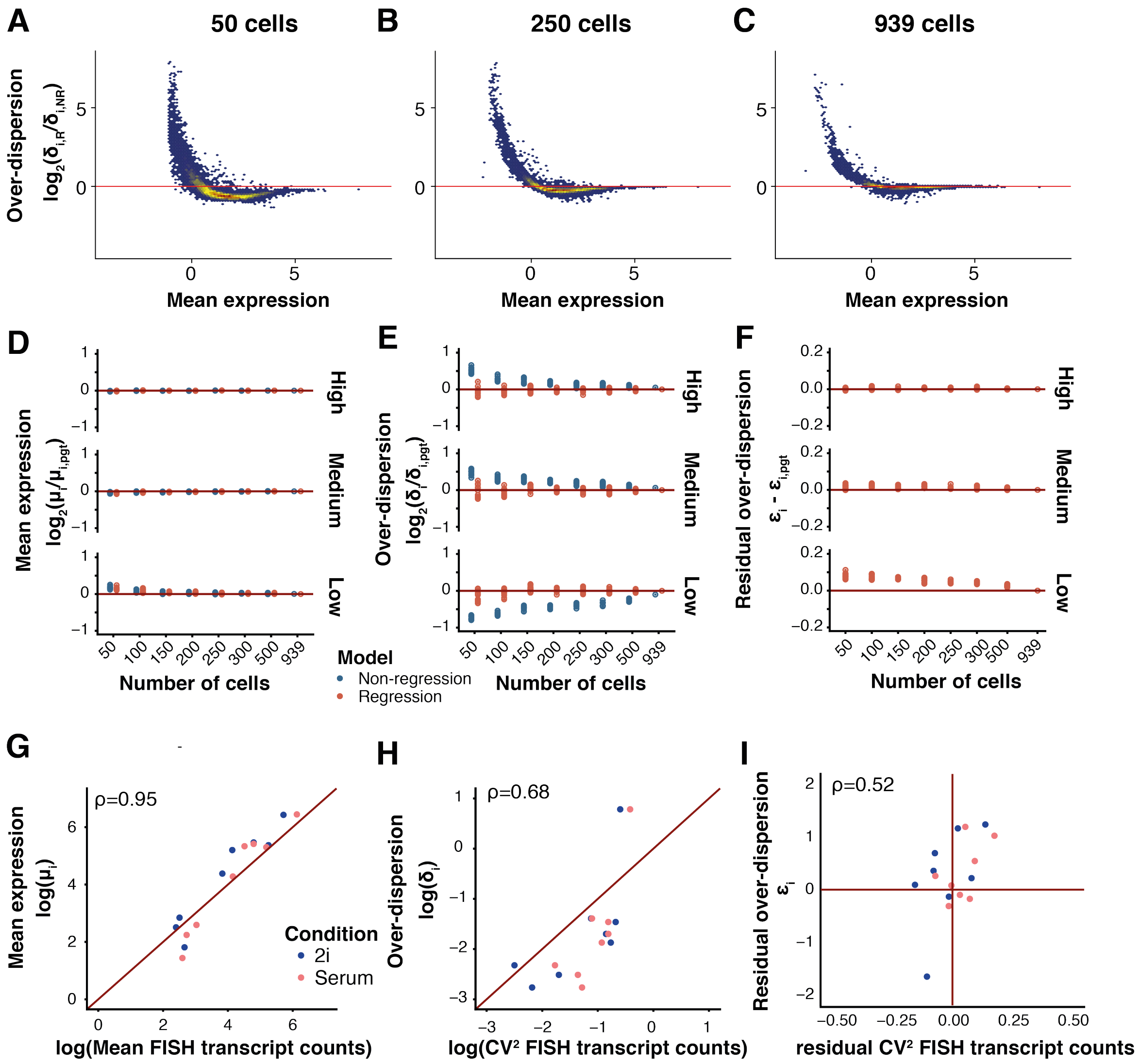

Figure S3: Stability of posterior estimates for gene-specific parameters (Related to Figure 3)

(A-C) The regression and non-regression BASiCS models were used to estimate gene-specific model parameters for three difference sample sizes: (A) 50 cells, (B) 250 cells and (C) 939 cells. The cells used in (A) and (B) were randomly sampled from the full population of 939 pyramidal CA1 neurons (Zeisel et al., 2015) (see **STAR Methods**). The $\log_2$ fold change in over-dispersion estimates between the regression and non-regression BASiCS models (vertical axis) is plotted against overall mean expression (horizontal axis). The colour code within the scatterplots is used to represent areas with high (yellow/red) and low (blue) concentration of genes.

Figure S3: Stability of posterior estimates for gene-specific parameters (Related to Figure 3, continued).
(D-F) The regression (orange) and non-regression (blue) BASiCS models were used to estimate gene-specific model parameters for lowly (lower panels), medium (mid panels) and highly (upper panels) expressed genes across populations with varying numbers of cells. These were generated by randomly sub-sampling cells from a population of 939 pyramidal CA1 neurons (see **STAR Methods** and Zeisel et al., 2015). For 10 sub-sampling experiments, parameter estimates were compared against a *pseudo* ground truth (pgt). The latter is defined as the parameter estimates obtained for the full population of 939 cells using the regression BASiCS model. For each sub-sampling experiment, gene-specific $\log_2$ fold changes ($\log_2(\mu_i/\mu_{i,pgt})$ and $\log_2(\delta_i/\delta_{i,pgt})$) and distances ($\epsilon_i - \epsilon_{i,pgt}$) between the estimates and the pgt were computed. For visualisation purposes, the medians across genes for each sub-sampling experiment are presented.
(G-I) Matched scRNAseq and smFISH data measured on mouse embryonic stem cells grown in 2i and serum media (see **STAR Methods** and Grün et al., 2014) was used to validate the performance of the regression BASiCS model. Gene-specific parameter estimates obtained by the regression BASiCS model were compared against empirical estimates calculated based on smFISH data. This comparison includes 8 genes, measured in both conditions. Pearson's correlation is indicated for each comparison. (G) Estimates of mean expression parameters μ_i (log-scale) are plotted against mean transcript count (smFISH). (H) Estimates of over-dispersion parameters δ_i (log-scale) are plotted against the squared coefficient of variation (CV^2) of transcript counts (smFISH). (I) Estimates for residual over-dispersion parameters ϵ_i are compared against residual estimates of variability estimated for the smFISH data (see **STAR Methods**).

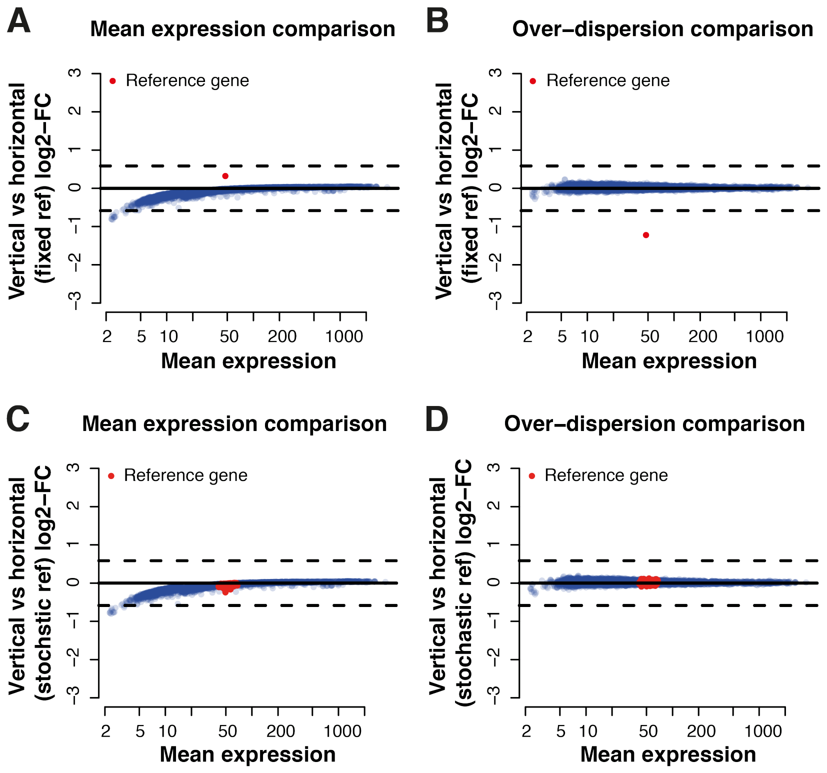

Figure S4: Estimates of genes-specific model parameters as computed by the vertical and horizontal data integration implementation of BASiCS (Related to Figure 4).
Comparison between the vertical (spikes) and horizontal (no spikes) data integration implementations of BASiCS using a dataset of mouse embryonic stem cells grown in a 2i medium (see **STAR Methods** and Grün et al., 2014). Dashed horizontal lines located at $\pm\log_2(1.5)$ indicate the default minimum tolerance $\log_2$ fold change threshold τ_0 used for differential testing. For details on the implementation see **STAR Methods**.
(A)-(B) An arbitrarily chosen reference gene is used to capture the identifiability restriction. Comparison in terms of posterior estimates for (A) mean expression parameters μ_i and (B) over-dispersion parameters δ_i across all genes.
(C)-(D) A stochastic reference choice is implemented by randomly selecting a different reference gene at each iteration of the MCMC algorithm. As a default, only the 10% of genes whose mean expression estimate is closest to μ_0 (based on Lun et al. (2016) normalization) are used as candidate reference genes. Comparison in terms of posterior estimates for (C) mean expression parameters μ_i and (D) over-dispersion parameters δ_i across all genes.

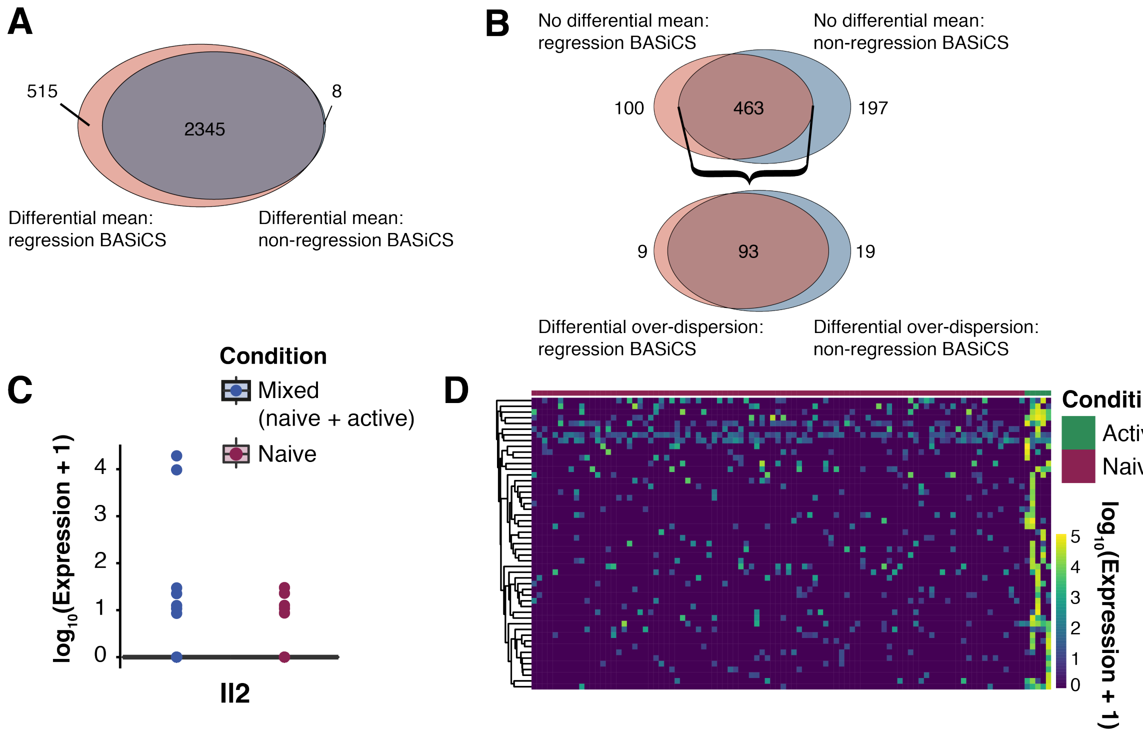

Figure S5: Extended analysis for CD4$^+$ T cell activation data (Related to Figure 5). (A)-(B) Results of differential testing between naive and activated CD4$^+$ T cells were compared between the regression and non-regression BASiCS models. As in Martinez-Jimenez et al. (2017), genes with low mean expression ($\mu_i < 50$) in both conditions were excluded from testing (see **STAR Methods**).

(A) Overlap of differentially expressed genes (mean) using a minimum tolerance threshold $\tau_0 = 2$ obtained using the regression and non-regression BASiCS models (EFDR = 10%).

(B) Upper panel: overlap of genes detected as non-differentially expressed using an stringent minimum tolerance threshold $\tau_0 = 0$ obtained using the regression and non-regression BASiCS models (EFDR = 10%). Lower panel: overlap of differentially over-dispersed genes using a minimum tolerance threshold $\omega_0 = \log_2(1.5)$ obtained using the regression and non-regression BASiCS models for the 463 genes detected as non-differentially expressed by both models (EFDR = 10%).

(C) Distribution of denoised expression counts for *Il2* in a population of naive CD4$^+$ T cells (red) and the mixture population representing a mix of 93 naive and 5 activated CD4$^+$ T cells (blue). Each dot represents a single cell.

(D) For the mixed population (93 naive and 5 activated CD4$^+$ T cells), heatmap of denoised expression counts for all genes highlighted to have increased mean expression and increased variability in the mixed population.

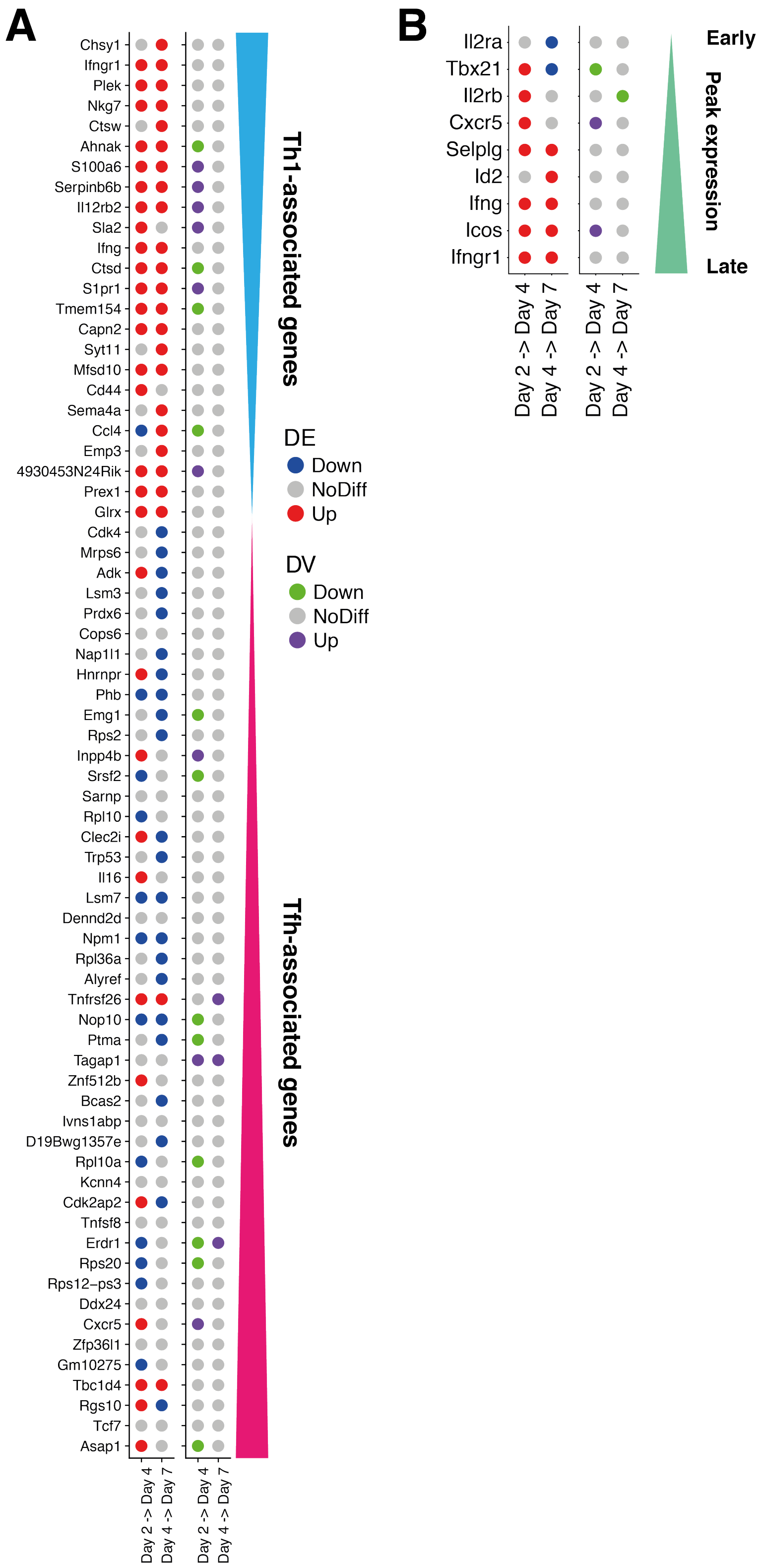

Figure S6: Differential regulation of Th1- and Tfh-associated genes across the differentiation process (Related to Figure 6).

Figure S6: Differential regulation of Th1- and Tfh-associated genes across the differentiation process (Related to Figure 6, continued).
Differential mean expression testing (minimum tolerance threshold $\tau_0 = 1$) and differential residual over-dispersion testing (minimum tolerance threshold $\psi_0 = 0.41$) was performed on cell populations between day 2 and day 4 as well as day 4 and day 7 controlling the EFDR to 10%. Genes that increase in expression over time are marked with a red dot while genes that decrease in expression over time are marked with a blue dot. Similarly, genes that increase in variability over time are marked in purple while genes that decrease in variability over time are marked in green. Only genes that pass filtering (see **STAR Methods**) are visualized.
(A) Differential testing results are visualized for Th1- and Tfh-associated genes taken from Figure 3E in Lönnberg et al. (2017). Genes are ordered based on their correlation with the Th1 trend assignment (top to bottom) or their correlation to Tfh trend assignment (bottom to top).
(B) Differential testing results are visualized for important genes during $CD4^+$ T cell differentiation (taken from Figure 5A in Lönnberg et al., 2017). Genes were ordered based on their peak expression point in pseudotime as defined by Lönnberg et al. (2017).

Supplemental Tables

Table S1: Datasets used in this study. Related to Figure 2, Figure S2 and STAR Methods.

For each of the datasets analysed in this study: number of cells (2nd column), number of genes (biological + technical spike-ins, 3rd column), number of batches (4th column), type of data acquisition system (5th column), information of which model (spikes = horizontal data integration, non-spikes = vertical data integration) was used to analyse the data (6th column), information of whether the data was generated using unique molecular identifiers (UMIs, 7th column) and the reference to the original study (8th column) are provided.

Dataset	# cells	# genes	# batches	scRNAseq protocol	BASiCS model	UMIs	Reference
Young naive $CD4^+$ T cells	93	10553	2	Fluidigm C1	Spikes	No	(Martinez-Jimenez et al., 2017)
Young active $CD4^+$ T cells	53	10553	2	Fluidigm C1	Spikes	No	(Martinez-Jimenez et al., 2017)
Microglia cells	98	10687	1	Fluidigm C1	Spikes	Yes	(Zeisel et al., 2015)
CA1 pyramidal neurons	948	10687	1	Fluidigm C1	Spikes	Yes	(Zeisel et al., 2015)
Malaria infected $CD4^+$ T cells day 2	89	7899	2	Fluidigm C1	Spikes	No	(Lönnberg et al., 2017)
Malaria infected $CD4^+$ T cells day 4	133	7899	2	Fluidigm C1	Spikes	No	(Lönnberg et al., 2017)
Malaria infected $CD4^+$ T cells day 7	64	7899	1	Fluidigm C1	Spikes	No	(Lönnberg et al., 2017)
Dictyostelium cells day 0	131	10738	3	Fluidigm C1	Spikes	No	(Antolovic et al., 2017)
Pool-split RNA 2i medium	76	8924	2	CEL-Seq	Spikes	Yes	(Grün et al., 2014)
Mouse embryonic stem cells 2i medium	74	8924	2	CEL-Seq	Spikes + Non-spikes	Yes	(Grün et al., 2014)
Pool-split RNA serum medium	56	8924	2	CEL-Seq	Spikes	Yes	(Grün et al., 2014)
Mouse embryonic stem cells serum medium	52	8924	2	CEL-Seq	Spikes	Yes	(Grün et al., 2014)
